# Direct and indirect mortality impacts of the COVID-19 pandemic in the US, March 2020-April 2021

**DOI:** 10.1101/2022.02.10.22270721

**Published:** 2022-02-15

**Authors:** Wha-Eum Lee, Sang Woo Park, Daniel M Weinberger, Donald Olson, Lone Simonsen, Bryan T. Grenfell, Cécile Viboud

**Affiliations:** 1 Department of Ecology and Evolutionary Biology, Princeton University, Princeton, USA, 08544; 2 School of Public Health, Yale University; 3 Department of Health and Mental Hygiene, New York, New York; 4 Department of Science and Environment, Roskilde University, Denmark; 5 Princeton School of Public Affairs, Princeton University, Princeton, USA, 08544; 6 Division of International Epidemiology and Population Studies, Fogarty International Center, National Institutes of Health, Bethesda, MD, USA. 20892

## Abstract

Excess mortality studies provide crucial information regarding the health burden of pandemics and other large-scale events. Here, we used time series approaches to separate the direct contribution of SARS-CoV-2 infections on mortality from the indirect consequences of pandemic interventions and behavior changes in the United States. We estimated deaths occurring in excess of seasonal baselines stratified by state, age, week and cause (all causes, COVID-19 and respiratory diseases, Alzheimer’s disease, cancer, cerebrovascular disease, diabetes, heart disease, and external causes, including suicides, opioids, accidents) from March 1, 2020 to April 30, 2021. Our estimates of COVID-19 excess deaths were highly correlated with SARS-CoV-2 serology, lending support to our approach. Over the study period, we estimate an excess of 666,000 (95% Confidence Interval (CI) 556000, 774000) all-cause deaths, of which 90% could be attributed to the direct impact of SARS-CoV-2 infection, and 78% were reflected in official COVID-19 statistics. Mortality from all disease conditions rose during the pandemic, except for cancer. The largest direct impacts of the pandemic were seen in mortality from diabetes, Alzheimer’s, and heart diseases, and in age groups over 65 years. In contrast, the largest indirect consequences of the pandemic were seen in deaths from external causes, which increased by 45,300 (95% CI 30,800, 59,500) and were statistically linked to the intensity of non-pharmaceutical interventions. Within this category, increases were most pronounced in mortality from accidents and injuries, drug overdoses, and assaults and homicides, while the rate of death from suicides remained stable. Younger age groups suffered the brunt of these indirect effects. Overall, on a national scale, the largest consequences of the COVID-19 pandemic are attributable to the direct impact of SARS-CoV-2 infections; yet, the secondary impacts dominate among younger age groups, in periods of stricter interventions, and in mortality from external causes. Further research on the drivers of indirect mortality is warranted to optimize interventions in future pandemics.

## Introduction

As the official death toll of the coronavirus disease 2019 (COVID-19) continues to grow, the full impacts of the pandemic on a range of conditions remain debated. In the United States (US), over 74 million confirmed cases and 887,000 official deaths were reported as of February 1, 2022 (Johns Hopkins University, 2022). A pandemic of the magnitude of COVID-19 has secondary effects on unrelated health conditions; for instance, non-COVID-19 deaths increased in Spring 2020 at the height of the first wave in part due to avoidance of the healthcare system (Sharma et al., 2021; Woolf et al., 2020).

Excess mortality approaches have been used for over a century to capture the full scope of large-scale infectious disease events, heatwaves, and earthquakes, by measuring the rise in mortality over a historical baseline (Serfling, 1963; Weinberger et al., 2020). In the early phase of the pandemic, these approaches highlighted substantial underestimation in official statistics of COVID-19 deaths due to limited viral testing (Weinberger et al., 2020). More recent analyses have examined excess mortality patterns for specific causes of death by age and socio-demographic groups and have compared the COVID-19 death toll between countries (Banerjee et al., 2020, p. 19; Islam et al., 2021; Karlinsky & Kobak, 2021; Mena et al., 2021; Rossen, 2021; Woolf et al., 2021). Yet, it remains a challenge to separate the direct and indirect impacts of the pandemic. The direct impacts of the virus include potential effects on deaths ascribed to chronic conditions; for instance, death in a diabetic patient could have been triggered by SARS-CoV-2 infection and be coded as diabetes. The indirect impacts of the pandemic include avoidance of the healthcare system for treatment of acute conditions and for management of underlying chronic conditions, stressed healthcare systems in period of high COVID19 incidence, and societal disruptions (Sharma et al., 2021). In particular, the indirect impacts of the pandemic on mental health, violence, and addiction remain debated, with potentially large impacts on mortality (Faust, Du, et al., 2021; Faust, Shah, et al., 2021).

There is substantial heterogeneity in the trajectory of the COVID-19 pandemic and public health measures across the US, providing an opportunity to separate the contributions of viral infection on mortality from that of pandemic interventions, in a large country with homogenous death ascertainment. Here, we use time series approaches to separate the direct consequences of SARS-CoV-2 infection on age- state- and cause-specific mortality from the indirect effects of the pandemic. Our analyses cover three large waves of infections from March 2020 to April, 2021 in the US. We also compare our excess mortality estimates with serology (Asten et al., 2021; Verity et al., 2020) and explore between-state variation in SARS-CoV-2 infection fatality rates. Our analyses contrast the direct and indirect effects of the pandemic on several chronic conditions and shed light on the long-lasting consequences on violence and overdoses.

## Data and Methods

### Mortality Data

We obtained weekly mortality counts from the National Center for Health Statistics (NCHS) for the period August 1, 2014– April 30, 2021; we included 2014–2019 data to construct robust historical model baselines (Centers for Disease Control and Prevention, 2021a, 2021b). Data were stratified by state, 6 age groups (all ages, under 25 years, 25–44, 45–64, 65–74, 75–84, and over 85), and 8 underlying mortality causes (all causes, respiratory conditions, Alzheimer’s disease, cancer, cerebrovascular diseases, diabetes, heart disease, external causes; see supplement for disease codes). External causes include suicides, accidents, homicides, and opioids, among other conditions. We used aggregated mortality counts ascribed to COVID-19, influenza, pneumonia, and chronic lower respiratory diseases as an indicator of ‘respiratory mortality’, which was our most specific indicator of excess deaths directly caused by SARS-CoV-2 infection. We compiled weekly deaths with any mention of COVID-19 anywhere in the death certificate and considered those to be the official COVID-19 statistics (Centers for Disease Control and Prevention, 2021c).

To further explore patterns in external mortality causes, which include a range of conditions, we obtained additional monthly data by subcategories of deaths, including suicides, assaults and homicides, drug overdoses, accidents and unintentional injuries, and motor vehicle accidents (a subset of accidents) (Centers for Disease Control and Prevention, 2021d, 2021e). We also downloaded monthly deaths from external causes combined by age and region (Centers for Disease Control and Prevention, 2021f). External causes of deaths are typically released several months after other conditions and detailed data were unavailable at a weekly resolution.

### Other datasets

Age- and state-specific population estimates were obtained from CDC (Centers for Disease Control and Prevention, 2021g) and used to calculate mortality rates. To validate our excess mortality approach, we compared our mortality estimates against CDC state-specific seroprevalence surveys (Centers for Disease Control and Prevention, 2021h). We used data on the proportion of the population with SARS-CoV-2 antibodies to the nucleocapsid by late April 2021 to compare with our excess death estimates at the end of April 2021, given a similar delay between infection and death and infection and rise in antibodies. As the nucleocapsid is not a component of the vaccines used in the US, the serologic assay captures natural infections. We used our comparison of excess mortality against serology to estimate the infection fatality rate. We ran sensitivity analyses on the maximum seroprevalence reported during the study period, rather than seroprevalence at the end of the study period, to account for potential waning of natural immunity.

To adjust excess mortality models for the contribution of influenza, we downloaded weekly surveillance data from CDC FluView (Rudis et al., 2021). Finally, to evaluate the putative impacts of public health interventions on cause-specific mortality, we compiled the health containment index from the COVID-19 government response tracker which measures the strength of interventions by week and state (Oxford University, 2021).

All data used in the analysis were publicly available and exempt from human subjects review; data and code have been posted in a GitHub repository that will be publicly released upon publication.

### Analytic Approach

#### Excess Mortality Models

Before running mortality models, we adjusted mortality counts for reporting delays using a modified NobBS package in R (McGough et al., 2020). Even though there was little reporting delay for the data presented in this study, we wanted to ensure comparability with prior work (Weinberger et al., 2020). After adjustment, we applied seasonal regression models to weekly mortality rates in the pre-pandemic period, August 1, 2014–March 1, 2020, and estimated the baselines for each mortality cause, age group, and state (Goldstein et al., 2012; Weinberger et al., 2020; and supplement for more details). The model included harmonic terms for seasonality, time trends, and a proxy for weekly influenza incidence. We fitted the model to data until March 1, 2020 and projected the baseline forward until April 30, 2021. We estimated weekly excess mortality by subtracting the predicted baseline from the observed mortality that week; total excess mortality was the sum of weekly excesses (positive or negative) during March 1, 2020 – April 30, 2021. We used block bootstraps to generate 95% prediction intervals and obtain uncertainty intervals on estimates. For context, we also estimated excess mortality for the severe 2017/2018 influenza season.

We ran cause-specific excess mortality analyses nationally and for states that had sufficient mortality counts, as weekly death counts below 10 were blanked due to privacy concerns. States missing more than 2 weeks of data between March 2020 and April 2021 were excluded from the corresponding analyses. We ran respiratory excess mortality analyses for 16 states and non-respiratory analyses for 33 states (see Supplement for full list).

#### Estimation of direct and indirect pandemic impacts

To assess the direct and indirect impacts of the pandemic on mortality, we performed several correlation and regression analyses evaluating the trajectory of different causes of deaths by age and geography, building on earlier work on the 1968 influenza pandemic (Reichert et al., 2004) and COVID-19 (Sharma et al., 2021) (Details in the Supplement). First, we tested whether weekly non-respiratory excess mortality became more correlated with respiratory excess mortality during March 2020–April 2021, compared to the pre-pandemic period. This would signal a direct but undetected effect of COVID-19 on non-respiratory mortality. Second, we assessed whether states that experienced high COVID-19 mortality concomitantly experienced high mortality from other causes during the pandemic. We used our respiratory excess mortality indicator, and the official COVID-19 death toll, as complementary measures of COVID-19 mortality. Third, to quantify the relative contributions of SARS-CoV-2 infection (direct impact) and non-pharmaceutical interventions (indirect impact) on mortality, we regressed weekly cause- and age-specific excess mortality against COVID-19 deaths and the COVID-19 containment index, after exploring different lags between predictors and outcomes. Regression models were run nationally and at the state level. Uncertainty in weekly excess mortality estimates was propagated into the regression models (Supplement).

#### Validation of excess deaths based on serology; estimation of Infection Fatality Rate (IFR)

Since deaths are ultimately the result of infections, serology can provide a validation of excess mortality estimates for mortality indicators that are specific of SARS-CoV-2. To test the validity of the excess mortality approach, we regressed cumulative excess respiratory mortality rates against SARS-CoV-2 seroprevalence estimates at the state level, in a model without intercept since there should be a direct correspondence between rates of infection and death. We repeated this analysis with all-cause excess mortality, official COVID-19 deaths, and excess mortality in individuals over 65 years as the outcome variable. These analyses provided both a statistical validation of our excess mortality approach and an opportunity to estimate the IFR, which is the slope of these regressions. We propagated the errors obtained in excess mortality and seroprevalence estimates into IFR estimates (Supplement).

## Results

### Overall mortality patterns

**Figure 1:**
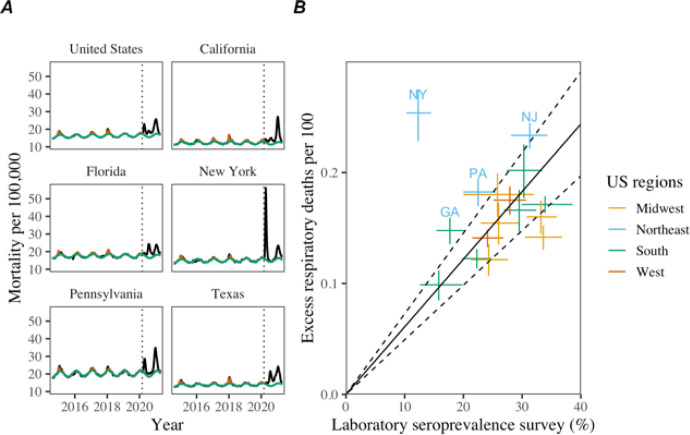


Across the US from March 1, 2020— April 30, 2021, there were 519,320 deaths officially attributed to COVID-19. During the same period, we estimate 507000 (95% Confidence Intervals (CI) 487000, 526000) excess respiratory deaths and 666,000 (95% CI 556000, 774000) excess deaths due to all-cause (Table 1). National mortality patterns comprise three waves from March 1–June 20, 202; June 21–September 19, 2020; and September 20, 2020 to April 30, 2021, with varying timing and intensity by state (Figure 1A and S1–S8). The first wave was concentrated in Northeastern states, while the Southern and Western states showed larger mortality increases during the second and third waves.

Excess respiratory mortality showed significant, positive correlation with seroprevalence surveys in each state (Figure 1B). Seroprevalence estimates ranged between 4.7 and 28% at the end of April 2021, with a population-weighted national seroprevalence of 22.8%. New York experienced higher than predicted excess mortality with respect to the reported serologic infection rates. This remained true in sensitivity analyses based on the maximum reported seroprevalence at any time point of the study period. The nationwide infection fatality rate (IFR) was estimated at 0.61% (0.49 – 0.73) based on excess respiratory mortality and 0.86% (0.72 – 0.99) based on all-cause excess mortality (Figure S9). Use of official COVID-19 deaths determined an IFR of 0.72% (0.62 – 0.81); interestingly, official COVID-19 deaths did not align with serology data as well as excess respiratory deaths (Figure S9). The IFR was significantly higher in individuals over 65 years, estimated at 5.7% (4.7–6.8).

By April 2021, the COVID-19 pandemic had caused significantly higher mortality compared to previous seasonal influenza outbreaks in the US. Respiratory excess mortality during COVID-19 exceeded the impact of the severe 2017/2018 influenza season 8.2 times (range across states 4.7–28.3).

### Direct and indirect pandemic impacts by cause of death

**Figure 2.**
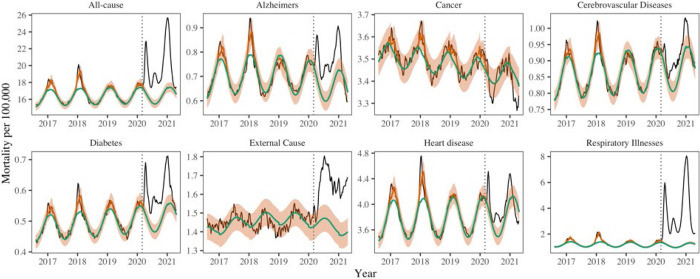
National mortality rate (per 100,000) for 8 causes of death. The black line shows observed data, the green line shows seasonal baseline, the orange shading the 95% CI on the seasonal baseline, and the red line shows model predictions with seasonal variation and influenza circulation. The dotted red lines show the upper and lower 95% confidence intervals. Excess mortality attributed to the COVID-19 pandemic is defined as the area between the black and green line from March 1, 2020. The dotted vertical line marks the start of the pandemic on March 1, 2020.

Excess mortality increased during the pandemic for 6 of the 7 non-respiratory conditions studied, although the timing and intensity of excess mortality varied by disease (Table 2, Figure 2). Cancer mortality was the only mortality condition that did not increase during the pandemic. Cancer deaths have remained below historic levels since March 2020, although cumulative weekly departures from baseline were not significantly different from zero (Table 2). In contrast, mortality from Alzheimer’s, diabetes, and heart disease rose during the pandemic, with the trajectory of excess mortality matching that of respiratory mortality in the 3 pandemic waves (Figures 2 for national patterns, and Figures S3–S8 for state-specific data). Across these causes of death, the first excess mortality peaks occurred within one week of the first respiratory mortality peak on April 18, 2020, and synchronicity between mortality causes was most evident in New York and New Jersey in the first wave. The third wave had a consistently larger peaks across diseases (Figure S10).

**Figure 3:**
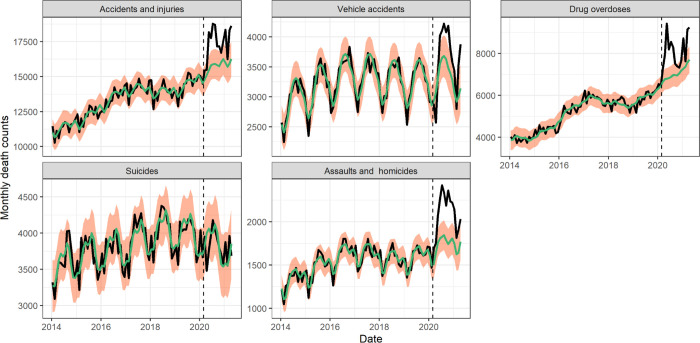
National monthly deaths by subcategory of external causes of death from January 1, 2014 to April 1, 2021. The black line shows observed data, the green line shows seasonal baseline, the orange shading represents the 95% CI on the seasonal baseline. The dotted vertical line marks the start of the pandemic on March 1, 2020.

We found a significant rise in deaths from external causes during the pandemic period March 2020–April 2021, corresponding to 45,300 (30,800, 59,500) excess deaths nationally (Figure 2). The largest excess mortality rates from external causes were estimated in states that also had high baseline death rates from these conditions (Figure S11). The weekly trajectory of mortality from external causes did not align with that of respiratory mortality. We further analyzed subcategories of external causes that were available on a monthly resolution (Figure 3). The largest excess death tolls observed during this period were from accidents and injuries (23,800 (8,400–39,200), an 11% increase over baseline), drug overdoses (15,300 (7,500–23,100), a 15% increase) and assaults and homicides (5,100 (2,700–7,600), a 21% increase, Table 3). Overdoses were the first to peak in May 2020, followed by accidents and assaults in July 2020. In contrast, mortality from suicides remained within historic baselines throughout the COVID-19 period.

We saw evidence of increased synchronicity in multiple causes of deaths during COVID-19, which is a possible signature of the direct effects of SARS-CoV-2 infection on mortality from chronic conditions. During the period March 2020–April 2021, all-cause mortality became more correlated with excess deaths from underlying respiratory conditions as compared to historical patterns in 14 out of 16 states (Figure S12). States that experienced high cumulative excess respiratory deaths had concomitantly high excess mortality from all-causes (Spearman rho = 0.73, 95% CI: 0.44 – 0.90)), attesting to the large impact of COVID-19 on total mortality (Figure S13). Synchrony between excess deaths from underlying respiratory diseases and excess deaths from underlying chronic conditions increased during the pandemic in a subset of states (Figure S12), particularly for diabetes (n=8 states), heart diseases (n=5), cerebrovascular diseases and Alzheimer (n=4). Excess deaths recorded as cancer, and external causes of deaths, showed no change, or declining synchrony, with respiratory mortality during the pandemic.

Next, to quantify the direct and indirect impacts of the pandemic on different causes of death, we regressed weekly cause-specific excess mortality against COVID-19 intensity and the strength of non-pharmaceutical interventions. On a national level, 90% (95% CI, 82–99) of excess deaths from all-causes were directly attributable to COVID-19, while the proportion was 59% (95% CI, 42 – 76) for diabetes, 58% (95% CI: 30 – 86) for Alzheimer’s, and 97% (95% CI: 40–158%) for heart diseases (Table 2). The upper bound of the 95% confidence interval for heart diseases was above 100% (158%), suggesting that for every excess death from heart disease estimated by our model, up to 1.58 death from heart disease could be directly linked to SARS-CoV-2 infection.

We also found evidence for the indirect effects of the pandemic on external causes of deaths and cancer. Periods of more stringent interventions were statistically associated with elevated mortality from external causes, while stricter interventions coincided with a decline in cancer mortality (Table S1). Analyses of direct and indirect effects at the state level yielded similar findings, with COVID-19 explaining 76 % (95% CI, 32–100) of all-cause excess deaths (Table S2). We did not identify predictors of cause-specific mortality at the state level, possibly due to lack of power (Table S2).

### Direct and indirect pandemic impacts by age group

**Figure 4.**
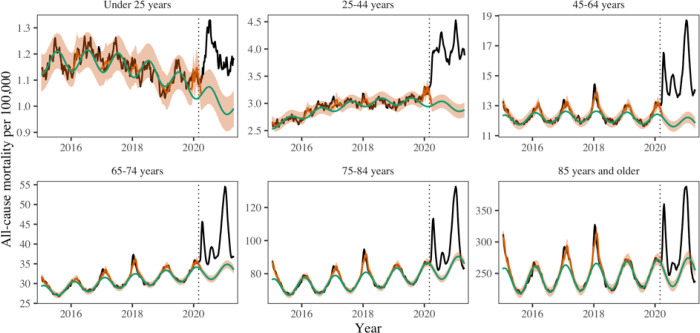
Age-specific all-cause mortality time-series graphs. The black line shows observed data, the green line shows seasonal baseline, the orange shading the 95% CI on the seasonal baseline, and the red line shows model predictions with seasonal variation and influenza circulation. The dotted red lines show the upper and lower 95% confidence intervals. Excess mortality attributed to the COVID-19 pandemic is defined as the area between the black and green line from March 1, 2020. The dotted vertical line marks the start of the pandemic on March 1, 2020.

The total burden and direct impacts of COVID-19 varied substantially by age (Figure 4, Table 4). Individuals 85 years and older had 155,100 (117300, 192100) excess all-cause deaths, accounting for 23% of excess deaths during the pandemic period. In contrast, individuals under 25 years and 25–44 years accounted for only 1.5% and 8.1% of pandemic excess mortality, representing 10,200 (6000, 14300) and 54,500 (47500, 61300) excess all-cause deaths.

Official COVID-19 statistics captured an increasing percentage of excess deaths with age, ranging from 11% (8–19%) in individuals under 25 years to over 100% in those over 85 years (Table 4). This age gradient is consistent with a larger direct effect of the pandemic in older age groups. We then used weekly regression of excess mortality on COVID19 activity and the strength of interventions to confirm these findings using a different approach and disentangle the direct and indirect pandemic effects. The COVID-19 term measuring the direct impact of viral infection was highest in older age groups and statistically significant in all age groups but those under 25 years. In contrast, the indirect impact of the pandemic measured by the intervention term was highest in youngest age groups, decreased with age, and lost significance in individuals above 65 years. This analysis indicates that deaths in younger ages rose in periods of stricter interventions, independently from the effect of SARS-CoV-2 circulation (Table S3). Finally, to better understand the interplay between indirect mortality in younger age groups and deaths from external causes, we visualized age-specific monthly statistics on external deaths. Excess deaths from external causes were concentrated in ages 15–44 years, with a notable elevation in May-July 2020 compared to historic baselines (Figure S14).

## Discussion

In this US study, we aim to disentangle the direct and indirect mortality impacts of the COVID-19 pandemic from March 2020–April 2021 using regression and synchronicity analyses. We find that 90% of the rise in all-cause mortality during this period can be statistically linked to SARS-CoV-2 infection, lending support to the predominance of the direct mortality consequences of the pandemic on a national scale. We also find a direct contribution of SARS-CoV-2 infections on chronic conditions such as Alzheimer’s, diabetes, cerebrovascular diseases, and heart diseases, which was missed in official statistics. Yet, analysis of mortality in children and young adults, and mortality from accidents and injuries, drug overdoses, assaults and homicides, paints a different picture. In these death categories, the mortality elevation observed during the pandemic period is statistically linked with interventions, supporting indirect pandemic effects rather than the direct consequences of SARS-CoV-2 infection. In contrast to other causes of deaths studied, cancer and suicides remained within baseline levels during the pandemic.

Perhaps the most striking finding of our study is the large mortality burden of the pandemic in individuals 25–44 years, with an estimated 54,500 (47,500, 61,300) excess deaths by April 2021. Only a quarter of these excess deaths are captured by official tallies of COVID-19 deaths. Accordingly, our regression analysis indicates that the majority of excess deaths in this age group are attributable to the indirect consequences of the pandemic. The trajectory of mortality in this age group is disjoint from the periods of intense COVID-19 circulation and statistically tied to the strength of interventions, supporting a possible detrimental effect of COVID-19 control measures beyond the initial lockdown period in Spring 2020. And even though children had a low absolute rate of excess mortality during the pandemic, only 11% was attributable to SARS-CoV-2 infection, while stricter interventions were associated with rising mortality in this age group. In contrast, we find that the predominant mortality impact of the pandemic in individuals over 65 years was associated with the direct consequences of SARS-CoV-2 infection. In a study of excess mortality in over 100 countries, Karlinsky and Kobak note that the direct mortality consequences of the pandemic typically predominate over the indirect consequences (Karlinsky & Kobak, 2021); however, they did not study age patterns. Our results support that the balance between direct and indirect effects is age-dependent and can skew towards indirect effects in the young, even in countries that experienced relatively high infections rates like the US.

Prior studies have shown that a decrease in emergency visits for diabetes, stroke, and myocardial infarctions in all age groups coincided with a rise in mortality for these conditions (Lange, 2020). Faust et al. estimated that 38% of deaths between 25 – 44 years were due to COVID-19 during March-July 2020, compared to 26% (23–30%) in our study that considers a much longer time period (Faust et al., 2020). Public health interventions, limited medical care, and behavioral changes (e.g., delays in seeking timely medical help due to fear of infection) (Bollmann et al., 2020; Kansagra et al., 2020; Mafham et al., 2020; Woolf et al., 2021) could have contributed to the surge in excess deaths unrelated to COVID-19 in young adults, resulting in a notable peak of mortality in summer 2020. In addition, we find that mortality from external causes remained elevated during May-August 2020 among young adults, likely driven by elevation in deaths from opioids, accidents, and assaults.

Mortality from external causes increased by 51,300 deaths (39700, 62700) from March 2020 – April 2021. There was a moderate correlation between excess mortality and pre-pandemic baseline rates for these causes (Figure S11), indicating that states with historically high death rates for suicide, drug overdose, and homicides experienced more prominent increases during the pandemic. The rise in mortality was most pronounced in assaults and homicide (21% over baseline, averaged over the pandemic period), followed by overdoses (15%) and accidents (11%). In contrast, mortality from suicides remained stable or below expectations. Prior work by Faust et al reported a decrease in suicide in several countries during March-July 2020, including in the US (10%) (Faust, Du, et al., 2021); here we show that suicide mortality remained stable throughout the rest of 2020. Further, Faust et al reported that deaths from overdoses and injuries increased during March-July 2020 (Faust, Du, et al., 2021). This is confirmed in our data, but we also show that the increase persisted in late 2020 and until the end of our study period in April 2021. Overall, of the 8 causes of death studied here, the indirect pandemic effects were statistically largest on external mortality causes.

Synchrony between respiratory mortality and other mortality causes was high during spring 2020, possibly due to poor SARS-CoV-2 test availability and guidelines to limit testing to just hospitalized cases. Many deaths in nursing homes or at-home during March-April 2020 were never tested, and they were recorded as known underlying conditions (i.e. heart disease, Alzheimer’s and diabetes) by default. In addition, Alzheimer patients typically live in nursing homes and may have been at increased risk of (untested) COVID-19 infection early in the pandemic. Interestingly, the correlation between excess mortality from respiratory diseases and Alzheimer increased in the winter 2020–2021 wave, signaling a persistent direct impact of COVID-19 on Alzheimer in a period where COVID-19 incidence and testing propensity were high.

We validated our excess mortality estimates against serology and assessed the IFR, a parameter notoriously difficult to measure. Our all-age estimate of 0.61% (0.49 – 0.73) is consistent with a recent meta-analysis (Meyerowitz-Katz & Merone, 2020) and aligns with an early study from China (0.66%; 95% CI: 0.39—1.33%) (Verity et al., 2020). A study of all-cause excess mortality in the Netherlands reports a substantially higher IFR (1%) (Asten et al., 2021); however, all-cause mortality is not specific to COVID-19 (our estimate based on all-cause mortality is higher at 0.86% (0.72 – 0.99)). IFR estimates based on official COVID-19 statistics were 15% higher than those based on excess respiratory mortality, yet the official statistics did not correlate with serology data as well as with our excess mortality estimates. Differences in COVID-19 death attribution by state could explain these findings. Overall, these analyses support the robustness of excess respiratory mortality as an indicator of COVID-19.

New York exhibited a notably high IFR. Several non-mutually exclusive factors could drive these findings, including a higher proportion of deaths among older individuals (aligned with the demography of New York state), large outbreaks in long-term care facilities, and lack of knowledge on management of severe patients early in the pandemic (Barnett et al., 2020). It is also worth noting that serosurveys conducted in April 2021 could underestimate cumulative SARS-CoV-2 infection rates in states that have experienced most of their infections in early 2020. Seroprevalence for New York state decreased from a peak of 23% in August 2020 to 13% in April 2021 in CDC data (Centers for Disease Control and Prevention, 2021f), which could be due to declining antibody titers (Buss et al., 2021) or sampling issues. To address this issue, we ran a sensitivity analysis using the maximum seroprevalence recorded over the study period rather than the seroprevalence at the end of the study period (Figure S9); New York remained an outlier in this analysis. Higher IFRs may be associated with healthcare systems working above capacity, as would have been the case in the first phase of the pandemic in New York.

The roll-out of a large SARS-CoV-2 vaccination campaign starting in late December 2020 in the US has had a major impact on rates of hospitalizations and deaths for COVID-19 in 2021. Accordingly, mortality from all causes and respiratory diseases declined continuously in the first months of 2021 and had just returned to baseline by the end of the study period in April 2021, before the arrival of new SARS-CoV-2 variants such as Delta and Omicron. It is interesting that at this particular point in the pandemic, in April 2021, mortality from cancer, Alzheimer and heart diseases was below baseline (negative excess mortality), with a similar phenomenon observed for all-cause mortality among individuals 75–84 and over 85 yrs. It is unlikely to be an artifact of reporting lags, as a reporting adjustment is built in the model, and reporting is 99% complete after 4 months. However, these negative excesses could signal a displacement of the mortality baseline, whereby frail individuals are harvested by large-scale infectious disease events or heatwaves, resulting in a decline in baseline mortality in the aftermath of an acute mortality event (Saha et al., 2013). Harvesting is also consistent with our regression analysis, where estimates of the direct impacts of COVID19 exceeded 100% in the two oldest age groups (albeit with broad confidence intervals, Table 4). This would be expected if baseline mortality was overestimated due to harvesting. Further, the age profile of COVID-19 severity risk dictates that older individuals would bear the strongest effects of harvesting, which is consistent with the US data. We can’t rule out the putative effect of SARS-CoV-2 vaccination on reducing baseline (non-COVID-19) mortality, although a direct biological mechanism is difficult to pinpoint.

Our study is subject to several limitations. First, mortality counts below the minimum cut-off value of 10 were suppressed due to privacy regulations. As a result, our age-specific analyses are restricted to larger states, and we could not assess the role of race/ethnicity. Prior work has shown important disparities in COVID-19 impact by race/ethnicity and economic status (Mena et al., 2021; Rossen, 2021) in the US and abroad. Second, official coding practices may have changed between states and through time based on SARS-CoV-2 testing availability, location of death, demographic factors, and comorbidities. Third, we find periods of negative excesses in cancer (throughout the pandemic), cardiovascular, and heart diseases (fall 2020), possibly due to changes in ascertainment of underlying cause of death (e.g. a death in a cancer patient with COVID-19 is ascribed to COVID-19) or harvesting (Saha et al., 2013). As discussed earlier, harvesting could also have affected estimates in oldest age groups. Similarly, we can’t account for changes in baseline respiratory mortality due to depressed circulation of endemic pathogens other than influenza. Finally, our study ends in April 2021 and does not capture a recrudescence of COVID19-related deaths due to the more transmissible Delta variant, primarily in states with low vaccine coverage, nor do we estimate the impact of the Omicron immune escape variant. As a result, our excess mortality estimates should be deemed conservative.

Pandemic excess mortality patterns have been heterogeneous globally (Islam et al., 2021; Karlinsky & Kobak, 2021; Kontis et al., 2020; Nørgaard et al., 2021). In a comprehensive analysis of mortality in 21 countries in Europe, New Zealand and Australia, official COVID-19 deaths accounted for an average of 77% (62%−93%) of all-cause excess deaths during the first wave of the pandemic March-May 2020 (Kontis et al., 2020). However, there was greater disconnect between estimates in hard-hit countries such as the UK, Spain, Italy, and Belgium (Kontis et al., 2020; Kontopantelis et al., 2021; Odone et al., 2021). In a study of over 100 countries, the ratio between excess mortality and official deaths was 1.6 on average, but went as high as 50 (e.g. Nicaragua) (Karlinsky & Kobak, 2021). The disconnect was primarily attributed to under-detection of COVID-19 rather than indirect effects, although indirect effects were not explicitly modeled. In Italy, the case fatality rate for acute myocardial infarction increased 3-fold during the first wave, while hospitalizations for these conditions decreased by 48% (Odone et al., 2021), suggesting that at least some of the excess deaths were indirect deaths. In the UK, cancer deaths increased about 10% at the height of the April 2020 lockdown; however, more recent fluctuations in cancer mortality remain unclear (Lai et al., 2020). Interestingly, in New Zealand, where control of COVID-19 has been remarkable, mortality was slightly but not significantly below baseline (Kontis et al., 2020). A similar finding was described in Russian provinces where a lockdown was implemented before the onset of COVID-19 (Kobak, 2021). This suggests that a lockdown without COVID-19 is neither preventing nor causing an appreciable number of deaths, although effects could be country-dependent. In the US, the direct impacts of the pandemic greatly outweigh the indirect consequences in all age data, but the reverse is true in children and young adults. Further work should concentrate on comparing the direct and indirect impact of COVID-19 in different countries over the same time period and using the same methodology.

## Conclusion

Here, we examined trends in cause-specific mortality across states and age groups to address the direct and indirect impacts of COVID-19 in the US. We find that ~90% of the total mortality elevation during the March 2020–April 2021 pandemic period is attributable to the direct impact of SARS-CoV-2 infection. There is however a large indirect impact of the pandemic in children and young adults, and on mortality from external causes, particularly from accidents, assaults, and overdoses. The indirect mortality impact of the pandemic is statistically linked to the strength of interventions. We also find an undetected contribution of SARS-CoV-2 infections on mortality from chronic conditions, such Alzheimer’s, diabetes, cerebrovascular conditions, and heart diseases, that persisted throughout winter 2020–2021. Our conclusions are based on ecologic analyses that are useful to generate hypotheses but do not prove causality. As more detailed mortality information become available with the release of death certificate data, it will be important to dissect the drivers of mortality among younger adults and certain ethnic groups, and understand how chronic conditions, violence, opioids and suicides intersect with large-scale infectious disease events and behavioral changes.

## Supplementary Material

1

## Figures and Tables

**Table 1.** Reported COVID-19 deaths, Compared with Excess Deaths from All-Causes and Respiratory Diseases: March 1, 2020 – April 30, 2021

Jurisdiction	Estimated excess all-cause deaths per 100000, (95% prediction interval)	Estimated excess all-cause deaths (95% prediction interval)	No. reported COVID-19 deaths*	Estimated excess respiratory deaths, (95% prediction interval)
*United States*	199 (166, 231)	666000 (556000, 774000)	519320	507000 (487000, 526000)
*Alabama*	333 (216, 445)	15700 (10200, 21000)	9913	9590 (8350, 10700)
*Mississippi*	315 (211, 416)	9590 (6410, 12700)	6471	NA
*New York*	296 (250, 342)	58000 (48900, 67000)	48775	50300 (45200, 54700)
*Louisiana*	291 (210, 370)	13700 (9920, 17500)	8684	NA
*New Jersey*	276 (226, 325)	26100 (21400, 30800)	21775	22500 (21300, 23600)
*South Carolina*	259 (177, 340)	12500 (8540, 16400)	8135	NA
*Arizona*	255 (203, 307)	21600 (17200, 26000)	14366	14900 (13700, 16000)
*Arkansas*	254 (151, 354)	7760 (4630, 10800)	5196	NA
*Oklahoma*	247 (153, 338)	9210 (5700, 12600)	7287	NA
*Tennessee*	233 (162, 304)	15800 (11000, 20600)	11591	11400 (10100, 12700)
*Texas*	233 (194, 270)	66700 (55600, 77400)	50094	49500 (47200, 51700)
*Ohio*	230 (156, 303)	26800 (18200, 35300)	19651	18800 (16900, 20600)
*Georgia*	222 (175, 269)	24100 (19000, 29200)	15855	16200 (14800, 17500)
*Pennsylvania*	221 (159, 282)	28300 (20300, 36100)	24291	23800 (22100, 25400)
*Kentucky*	218 (131, 303)	9630 (5790, 13400)	6391	NA
*Nevada*	214 (131, 295)	7390 (4520, 10200)	5205	NA
*California*	207 (171, 242)	87300 (72100, 102000)	59849	59700 (56100, 63000)
*Indiana*	202 (133, 269)	13400 (8820, 17900)	11684	12000 (10600, 13300)
*Maryland*	198 (138, 257)	12900 (8950, 16700)	8745	NA
*Connecticut*	197 (112, 280)	7250 (4110, 10300)	7489	NA
*Missouri*	195 (123, 266)	12100 (7600, 16500)	9801	9620 (8360, 10800)
*Illinois*	192 (145, 237)	25400 (19200, 31400)	19267	19000 (17500, 20500)
*Florida*	177 (135, 219)	41500 (31500, 51300)	29473	29200 (27000, 31300)
*Massachusetts*	161 (90, 230)	11000 (6170, 15800)	12133	NA
*Kansas*	159 (58.6, 257)	4590 (1690, 7420)	4385	NA
*Colorado*	157 (89.2, 222)	8270 (4710, 11700)	5574	NA
*Michigan*	156 (104, 208)	16700 (11100, 22200)	14932	13600 (12000, 15200)
*Virginia*	145 (92.3, 196)	12900 (8230, 17500)	9698	8940 (7640, 10100)
*Wisconsin*	138 (−43.2, 304)	8310 (−2590, 18200)	6610	NA
*Iowa*	137 (34.9, 238)	4150 (1050, 7190)	5309	NA
*Minnesota*	90 (25.4, 153)	5310 (1500, 9040)	6267	NA
*Oregon*	78 (−2.63, 156)	3320 (−112, 6660)	2073	NA
*Washington*	68.8 (11.4, 125)	5110 (846, 9280)	4577	NA

* As reported by National Center for Health Statistics. States are ordered from highest to lowest excess all-cause death rate per 100 000.

* No. of reported COVID −19 deaths (any death with COVID-19 as underlying cause) until April 30, 2021 as available on July 2, 2021, were obtained from the NCHS website^10^

**Table 2.** Estimation of the direct impacts of COVID-19 on non-respiratory conditions

Cause of Death	Estimated excess deaths (95% prediction interval)	% of excess deaths directly attributable to COVID-19*
All-Cause	666000 (556000, 774000)	90 (82, 99)
Alzheimer’s	17600 (8800, 26300)	58 (30, 86)
Diabetes	15800 (10500, 21000)	59 (42, 76)
Cancer	−4800 (−18400, 8600)	
Cerebrovascular Disease	8500 (1600, 15400)	50 (18, 83)
External Cause	45300 (30800, 59500)	
Heart Disease	27800 (−3100, 58000)	97 (40, 158)

* Shown only when COVID-19 is a significant covariate (p < 0.05); estimates are derived from a model where weekly excess mortality is regressed against weekly COVID-19 intensity and weekly interventions on a national scale, for the 60 weeks of March 2020 – April 2021.

**Table 3:** Excess mortality for different subcategories of external deaths during the COVID-19 pandemic period, March 2020 to April 2021.

Underlying cause of death	No of excess deaths (95% CI)	Ratio of excess deaths to baseline deaths (95% CI)**
Accidents (unintentional injuries)	23800 (8400, 39200)	0.11 (0.04, 0.18)
Motor vehicle accidents*	4300 (−700, −9300)	0.09 (−0.02, 0.2)
Drug overdoses	15300 (7500, 23100)	0.15 (0.08, 0.23
Assaults and homicides	5100 (2700, 7600)	0.21 (0.11, 0.31)
Suicides	−100 (−6000, 5800)	0 (−0.11, 0.11)

* Motor vehicle accidents is a subcategory of accidents.

** This should be interpreted as the percent increase over baseline. For instance, accidents increased 11% (95%CI, 4–18%) on average over baseline levels during the period March 2020–April 2021 (P<0.05).

**Table 3.** Excess all-cause deaths, official COVID-19 deaths, and direct contribution of COVID-19 to mortality, by age group.

Age	Estimated excess all-cause deaths, (95% prediction interval)	Estimated excess all-cause deaths per 100000, (95% prediction interval)	Official statistics on the no. reported COVID-19 deaths *	Percent of excess deaths coded as COVID-19 in official statistics (%)**	% of all-cause deaths directly attributed to COVID-19***
Under 25 years	10200 (6000, 14300)	9.73 (5.74, 13.6)	1127	11 (8, 19)	NA
25–44 years	54500 (47500, 61300)	60.8 (53.1, 68.5)	14177	26 (23, 30)	22 (16, 29)
45–64 years	150600 (132100, 168900)	180 (158, 201)	99594	66 (59, 75)	62 (56, 68)
65–74 years	152400 (133500, 171100)	461 (404, 517)	127094	83 (74, 95)	89 (83, 96)
75–84 years	139700 (115900, 163100)	840 (697, 980)	158202	113 (97, 136)	125 (114, 137)
85 years and older	155100 (117300, 192100)	2310 (1740, 2860)	173180	112 (90, 148)	135 (117, 154)

* Total mortality between March 1, 2020 – April 30, 2021 as available on July 5, 2021

* Death certificates have multiple causes of deaths listed; here COVID-19 can be listed anywhere in the death certificate

** Estimated as the proportion of excess all-cause deaths captured by official COVID-19 statistics (column 4 divided by column 2)

*** Regression estimates of the direct impact of COVID-19 on all-cause excess mortality, where all-cause excess mortality is regressed against interventions and COVID19 intensity each week.
