# Supplementary material for: Direct and indirect mortality impacts of the COVID-19 pandemic in the US, March 2020-April 2021
Source: medRxiv. 2022 Feb 15:2022.02.10.22270721. Preprint. [Version 1] doi: 10.1101/2022.02.10.22270721 (PMC8863161; doi:10.1101/2022.02.10.22270721)
Supplement: 1 [file NIHPP2022.02.10.22270721V1-supplement-1.pdf]

medRxiv preprint doi: https://doi.org/10.1101/2022.02.10.22270721; this version posted February 15, 2022. The copyright holder for this preprint (which was not certified by peer review) is the author/funder, who has granted medRxiv a license to display the preprint in perpetuity.
This article is a US Government work. It is not subject to copyright under 17 USC 105 and is also made available for use under a CC0 license.

Direct and indirect mortality impacts of COVID-19 in the US, March 2020-April 2021: Supplementary Online Content

Authors: Wha-Eum Lee[1], Sang Woo Park[1], Daniel Weinberger[2], Donald Olson[3], Lone Simonsen[4], Bryan T. Grenfell[1, 5], Cécile Viboud[6]

A. Supplemental Data and Methods

1. General approach

Our analysis relies on modeling of weekly trends in US death certificates compiled from the National Center for Health Statistics (NCHS) website [1]. The goal of the study is to estimate the direct mortality impact of COVID-19, which results from SARS-CoV-2 infection, from the indirect impacts of the pandemic which can be linked to societal or health-related changes brought about by the pandemic.

We define direct COVID-19 mortality as the sum of deaths confirmed and coded as COVID-19 as the underlying cause, deaths coded as another underlying primary cause but with COVID-19 as one of the contributing multiple causes, and deaths that were directly caused by SARS-CoV-2 infection but for which a COVID-19 code is not included in the death certificate due to misdiagnosis or lack of testing. The first two categories are included in official death tallies of COVID-19 (since a COVID-19 code appears in the death certificate), while the third category can only be estimated using excess mortality approaches.

We define the indirect mortality impact of the pandemic as the sum of deaths due to healthcare avoidance or inaccessibility, deaths due to conditions or events that are exacerbated by non-pharmaceutical interventions and other pandemic behavior, and would not have occurred otherwise (i.e. suicide, drug overdose, homicide, or a stressed healthcare system that is unable to treat conditions unrelated to SARS-CoV-2).

It is worth noting that circulation of multiple pathogens has plummeted due to social distancing interventions in 2020-2021,[2] and therefore the pandemic could have prevented a number of infectious disease deaths relative to historical expectations. Excess mortality approaches[3,4] will capture the net sum of these indirect impacts. These direct and indirect mortality pathways are not mutually exclusive and plausible over a wide range of conditions. For example, SARS-CoV-2 infection may trigger death in a patient with diabetes, which may be missed by testing, with no COVID-19 code listed on the death certificate. Concomitantly, a diabetic patient without a recent history of SARS-CoV-2 infection may turn away from the healthcare system at the height of the pandemic and die from lack of treatment. Our analysis attempts to separate these effects.

2. Mortality Data

a. Mortality conditions studied and states selected for further analysis:

We used the international classification of disease version-10 to retrieve deaths for the following 8 specific conditions: All-cause (deaths from any causes), Alzheimer's (G30), Cancer (C00-C97), Cerebrovascular diseases (I60-I69), Diabetes (E10 – E14), Heart disease (I00-I09, I11,I13,I20-I51), Respiratory Conditions (J09-J18, J40-J47, U071), External Cause (V01-Y89,

medRxiv preprint doi: https://doi.org/10.1101/2022.02.10.22270721; this version posted February 15, 2022. The copyright holder for this preprint (which was not certified by peer review) is the author/funder, who has granted medRxiv a license to display the preprint in perpetuity. This article is a US Government work. It is not subject to copyright under 17 USC 105 and is also made available for use under a CC0 license.

U01-U03). Deaths with any of these codes as the underlying cause of deaths were selected for analysis.

16 states were selected for further analysis of respiratory mortality because they had sufficient counts on a weekly basis: Alabama, Arizona, California, Florida, Georgia, Illinois, Indiana, Michigan, Missouri, New Jersey, New York, Ohio, Pennsylvania, Tennessee, Texas, Virginia. Weekly deaths count below 10 are blanked by NCHS for privacy reasons; states that had more than 2 weeks of blanked observations during the pandemic period were excluded. Respiratory mortality was our most restrictive mortality outcome; it is based on aggregation of deaths from pneumonia and influenza with deaths from other respiratory conditions. Pneumonia and influenza can be uncommon on a weekly basis in small states, especially in summer, and hence blanked observations are not uncommon in the state-level dataset.

We applied a similar reasoning to the 7 other causes of deaths that are unrelated to respiratory conditions. Death counts were more numerous for these conditions than for respiratory mortality. If a state had more than 2 blanked weeks for one of the conditions, it was excluded for analysis of the other conditions. The following 33 states were included for analysis of non-respiratory mortality: Alabama, Arizona, Arkansas, California, Colorado, Connecticut, Florida, Georgia, Illinois, Indiana, Iowa, Kansas, Kentucky, Louisiana, Maryland, Massachusetts, Michigan, Minnesota, Mississippi, Missouri, Nevada, New Jersey, New York, Ohio, Oklahoma, Oregon, Pennsylvania, South Carolina, Tennessee, Texas, Virginia, Washington, Wisconsin.

3. Analytical approach

a. Adjustment for reporting delays

Death counts are not finalized until several weeks after occurrence in the NCHS database. To estimate reporting delays, and for consistency with a prior analysis [3], we used a modified version of the NobBS package in R. The algorithm uses “snapshots" of data downloaded across different periods to infer reporting delays and the completeness of data at each reporting interval, which are then used to estimate true death counts. For this study, we used data from the NCHS website[1] downloaded every Friday for 25 weeks (July 17, 2020–January 22, 2021, with the exception of week 2020-10-02). This algorithm was used to adjust reporting delay for all-cause and cause-specific mortality, allowing reporting delays to vary by cause and state.

b. Weekly excess Mortality Model

We applied seasonal regression models to weekly cause- and age-specific mortality, inspired by prior work on COVID-19 [3,4]. Models included time trends, harmonic terms for seasonality, and terms for influenza circulation, following:

$$y_t = \beta_0 + \beta_1 t + \beta_2 t^2 + \beta_3 \sin\left(\frac{2\pi t}{52.17}\right) + \beta_4 \cos\left(\frac{2\pi t}{52.17}\right) + \sum_{i=1}^{6} \alpha_i x_{\{i,t\}} + e_t$$

where t = time
$\beta_0 = intercept$
β_1 and $\beta_2 = time\ trends\ coefficients$
β_3 and $\beta_4 = seasonal\ coefficients$

medRxiv preprint doi: https://doi.org/10.1101/2022.02.10.22270721; this version posted February 15, 2022. The copyright holder for this preprint (which was not certified by peer review) is the author/funder, who has granted medRxiv a license to display the preprint in perpetuity. This article is a US Government work. It is not subject to copyright under 17 USC 105 and is also made available for use under a CC0 license.

α_i = flu coefficients in season i
$x_{i,t}$ = influenza proxy at time t, season i
e_t=normally distributed error terms

The proxy for weekly influenza incidences was calculated by multiplying the weekly percentage of physician visits for influenza-like illness and weekly percentage of positive influenza tests, [4] which were obtained via CDC's FluView portal using the cdcfluview package in R (package version 0.9.1). We let the influenza coefficient vary each season to reflect a different mix of circulating subtypes, associated with different severities. Influenza data were not available for New Jersey and Florida; instead, we used data from New York state and HHS Region 4, respectively. Some states discontinue laboratory surveillance for influenza virus circulation during the summer months, so we replaced the missing summer weeks with zero. Weekly influenza incidences after March 1, 2020 were set to zero, in line with the minimal circulation of influenza reported in this time period. [4] Computation of confidence intervals aligns with prior work on COVID-19 excess mortality in the US [3].

March 1, 2020 was set as the start of the period of putative pandemic-related excess mortality. We fitted the model to data until March 1, 2020 and projected the baseline forward until April 30, 2021. We estimated weekly excess mortality related to the COVID-19 pandemic by subtracting the predicted baseline from the observed mortality that week. Total excess mortality for the pandemic period was defined as the sum of weekly excesses (positive or negative) during March 1 – April 30, 2021.

Of note, the impact of seasonal influenza was estimated by having an explicit flu coefficient in the excess respiratory model, while the COVID-19 impact was estimated by taking the difference between the observed mortality and baseline mortality during the pandemic period. Generally, this would be expected to inflate the impact of COVID-19, relative to influenza. But given that our COVID-19 estimates based on respiratory deaths align particularly well with official COVID-19 counts, and circulation of respiratory pathogens was minimal during this time period except for SARS-CoV-2[2], we believe that our comparison of excess respiratory deaths between COVID-19 and seasonal influenza is fair.

We also tested different link functions and error structures for the model and report the best fit to data here.

c. Monthly excess mortality model for subcategories of external deaths

To better understand the rise in external deaths during the pandemic, we applied a similar excess mortality approach to deaths from 5 subcategories of external deaths available at a monthly resolution[5,6]: all accidents, motor vehicle accidents, drug overdoses, assaults and homicides and suicides. Because these data were on a monthly time scale, and less stationary that the other mortality causes, we tried more flexible model formulations including spline terms for time trends and/or for seasonality. Based on AIC, a model with spline terms for time trends and seasonality provided the best fit to the data. We did not include a term for influenza as there is no biological reason for why influenza would affect external deaths. External cause of death data were not available by subcategory and age, but we had age-specific data for all external causes of death combined [6]. We visually explored these age-specific data as there was not enough information to fit time series models (Fig S14).

medRxiv preprint doi: https://doi.org/10.1101/2022.02.10.22270721; this version posted February 15, 2022. The copyright holder for this preprint (which was not certified by peer review) is the author/funder, who has granted medRxiv a license to display the preprint in perpetuity. This article is a US Government work. It is not subject to copyright under 17 USC 105 and is also made available for use under a CC0 license.

d. Estimation of the direct and indirect mortality impacts of the pandemic

We use a two-step approach to estimate the direct and indirect impacts of the pandemic. In the first step, we estimate the national weekly excess mortality for a given cause of death and age group, along with confidence intervals. In a second step, we regress weekly cause- and age-specific excess mortality against the weekly strength of non-pharmaceutical interventions (Oxford Health containment index[7]) and weekly COVID-19 activity (proxied by the weekly number of official tallies of COVID-19 deaths in NCHS data). The number of excess deaths from any cause directly attributable to COVID-19 is given by the regression coefficient for COVID-19 multiplied by the cumulative sum of the weekly COVID-19 predictor. A similar logic applies to the contribution of interventions on mortality. We explored potential delays between excess mortality and covariates (intervention measures and COVID-19 activity) using cross-correlation analysis (ccf function in R). We identified a lag of 4 weeks for interventions, and no lag for COVID-19, consistent across mortality outcomes and age groups.

To propagate the uncertainty in excess mortality estimates (response variable) between the first and second regression steps, we resampled the weekly excess mortality estimates based on their mean estimated values and standard deviation provided by the step 1 seasonal regression model, assuming a normal distribution. We sampled excess mortality 1000 times at each week (generating 1000 time series of excess mortality) and performed univariate and multivariate regression using COVID19 activity and interventions as covariates. Then, for each regression, we approximated the estimated slope coefficients and associated confidence intervals using normal distributions and sampled from those 100 times. This resulted in 100,000 slope estimates, from which we estimated the final confidence intervals for the effect of COVID19 and interventions on different age groups and causes. This method allowed us to account for the uncertainty in the response variable as well as the uncertainty in each fit.

We ran a similar approach to explore direct and indirect mortality effects in state- and cause-specific data. We regressed the cumulative excess mortality rate for the March 2020-April 2021 pandemic period for a given state and cause of death against average COVID19 official death rate, interventions, and cause-specific mortality baseline for the same period and state. The inclusion of a mortality baseline predictor was used to test the hypothesis that states that have high mortality for a given condition in typical years also fare worse during the pandemic. We propagated uncertainty in the response variable in the same way that we did for the weekly national estimates. For all states and causes of death, we ran univariate analyses and used AIC for variable selection in multivariate models.

Here we used a two-step approach to estimate direct and indirect mortality effects (Step 1: estimate weekly excess mortality and Step 2: regress weekly excess mortality against weekly COVID19 activity and weekly interventions). An alternative approach would be to run a single model, where weekly mortality rates are regressed against seasonal terms, time trends, flu activity, COVID-19 activity, and interventions. However, because there were only 60 pandemic weeks in our dataset, compared to 292 pre-pandemic weeks where the COVID19 and intervention coefficients are zero, that would be insufficient to get a robust estimate of the COVID-19 and intervention coefficients.

e. Estimation of the Infection Fatality Ratio

medRxiv preprint doi: https://doi.org/10.1101/2022.02.10.22270721; this version posted February 15, 2022. The copyright holder for this preprint (which was not certified by peer review) is the author/funder, who has granted medRxiv a license to display the preprint in perpetuity. This article is a US Government work. It is not subject to copyright under 17 USC 105 and is also made available for use under a CC0 license.

Without accounting for delays between infection and death, the infection fatality ratio (IFR) can be estimated by dividing the total number of deaths by the total number of infections. In other words, we have:

$$IFR = \frac{Total\ number\ of\ deaths}{Total\ number\ of\ infections},$$
$$= \frac{(Total\ number\ of\ deaths)/(Population\ size)}{(Total\ number\ of\ infections)/(Population\ size)},$$
$$= \frac{Excess\ respiratory\ deaths\ per\ 100\ people}{\%\ seroprevalence}$$

assuming that excess respiratory deaths provide a good estimate for deaths attributable to COVID-19. If we have information on excess deaths and serology in multiple states, we can regress these factors against each other, and the slope gives the average nationwide IFR when the intercept is set to zero (Figure 1). Given that the delay between seroconversion after infection is around 2 weeks, and the delay between infection and death is in the same order of magnitude, we use excess deaths until April 30th, 2021 in the numerator and serology for the last week of April 2021 in the denominator.

To propagate uncertainty from both the response variable (excess mortality) and covariate (seroprevalence) into IFR estimates, we used a similar approach as for the direct and indirect attribution model in the previous section. We resampled from the reported estimates of excess mortality and seroprevalence in each state, assuming normal distributions aligned with the reported 95% CI. Then, we drew 10,000 samples of excess mortality and seroprevalence in each state, and for each sample data set, we performed a linear regression and estimated the slope. Next, we drew 100 random samples for each of the 10,000 slope distributions. We calculated the confidence intervals of our IFR estimate by aggregating random samples of IFR estimates across all 100,000 sample data sets and taking 2.5% and 97.5% quantiles.

References

1. Weekly Counts of Deaths by State and Select Causes, 2019-2020. Accessed December 16, 2021. https://data.cdc.gov/NCHS/Weekly-Counts-of-Deaths-by-State-and-Select-Causes/muzy-jte6

2. Baker RE, Park SW, Yang W, Vecchi GA, Metcalf CJE, Grenfell BT. The impact of COVID-19 nonpharmaceutical interventions on the future dynamics of endemic infections. *Proc Natl Acad Sci U S A*. 2020;117(48):30547-30553. doi:10.1073/pnas.201318211

3. Weinberger DM, Chen J, Cohen T, et al. Estimation of Excess Deaths Associated With the COVID-19 Pandemic in the United States, March to May 2020. *JAMA Intern Med*. 2020;180(10):1336. doi:10.1001/jamainternmed.2020.3391

4. Goldstein E, Cobey S, Takahashi S, Miller JC, Lipsitch M. Predicting the Epidemic Sizes of Influenza A/H1N1, A/H3N2, and B: A Statistical Method. *PLOS Med*. 2011;8(7):e1001051. doi:10.1371/journal.pmed.1001051

medRxiv preprint doi: https://doi.org/10.1101/2022.02.10.22270721; this version posted February 15, 2022. The copyright holder for this preprint (which was not certified by peer review) is the author/funder, who has granted medRxiv a license to display the preprint in perpetuity.
This article is a US Government work. It is not subject to copyright under 17 USC 105 and is also made available for use under a CC0 license.

5. *Monthly Provisional Counts of Deaths by Select Causes, 2020-2021*. Accessed December 16, 2021. https://data.cdc.gov/NCHS/Monthly-Provisional-Counts-of-Deaths-by-Select-Cau/9dzk-mvmi

6. *AH Monthly Provisional Counts of Deaths by Age Group and HHS region for Select Causes of Death, 2019-2021*. Accessed December 16, 2021. https://data.cdc.gov/NCHS/AH-Monthly-Provisional-Counts-of-Deaths-by-Age-Gro/ezfr-g6hf

7. Oxford Covid-19 Government Response Tracker. GitHub. Accessed February 16, 2021. https://github.com/OxCGRT/USA-covid-policy

medRxiv preprint doi: https://doi.org/10.1101/2022.02.10.22270721; this version posted February 15, 2022. The copyright holder for this preprint
(which was not certified by peer review) is the author/funder, who has granted medRxiv a license to display the preprint in perpetuity.
This article is a US Government work. It is not subject to copyright under 17 USC 105 and is also made available
for use under a CC0 license.

Figure S1: Trends in weekly all-cause mortality, nationally and by state
Black lines show observed data. Green line shows the seasonal baseline. The red solid line shows seasonal variation accounting for influenza circulation. The orange shading shows the upper and lower 95% confidence intervals. The dotted vertical line marks March 1, 2020.

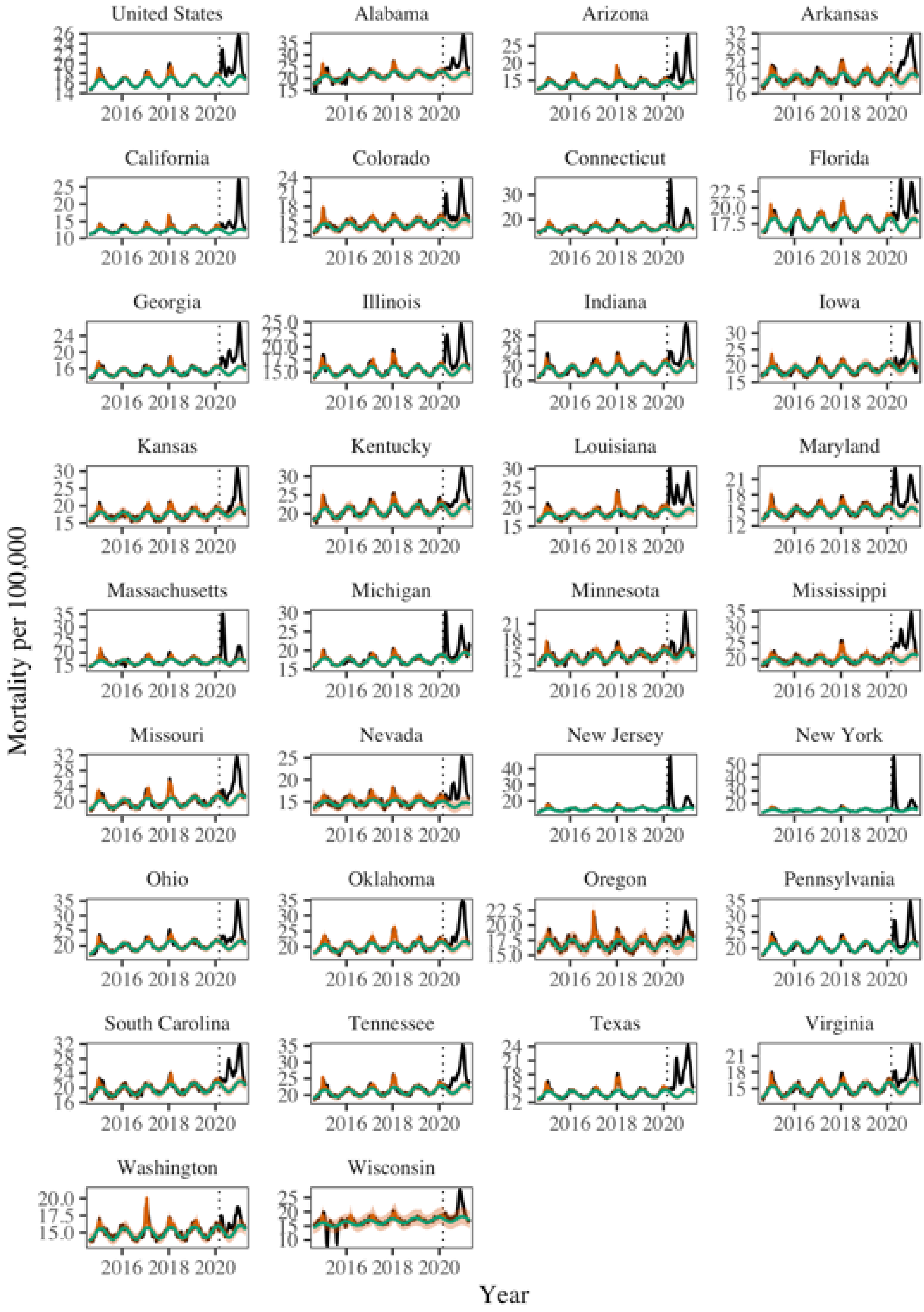

medRxiv preprint doi: https://doi.org/10.1101/2022.02.10.22270721; this version posted February 15, 2022. The copyright holder for this preprint (which was not certified by peer review) is the author/funder, who has granted medRxiv a license to display the preprint in perpetuity. This article is a US Government work. It is not subject to copyright under 17 USC 105 and is also made available for use under a CC0 license.

Figure S2: Trends in weekly Alzheimer mortality, nationally and by state. Legend as in Figure S1

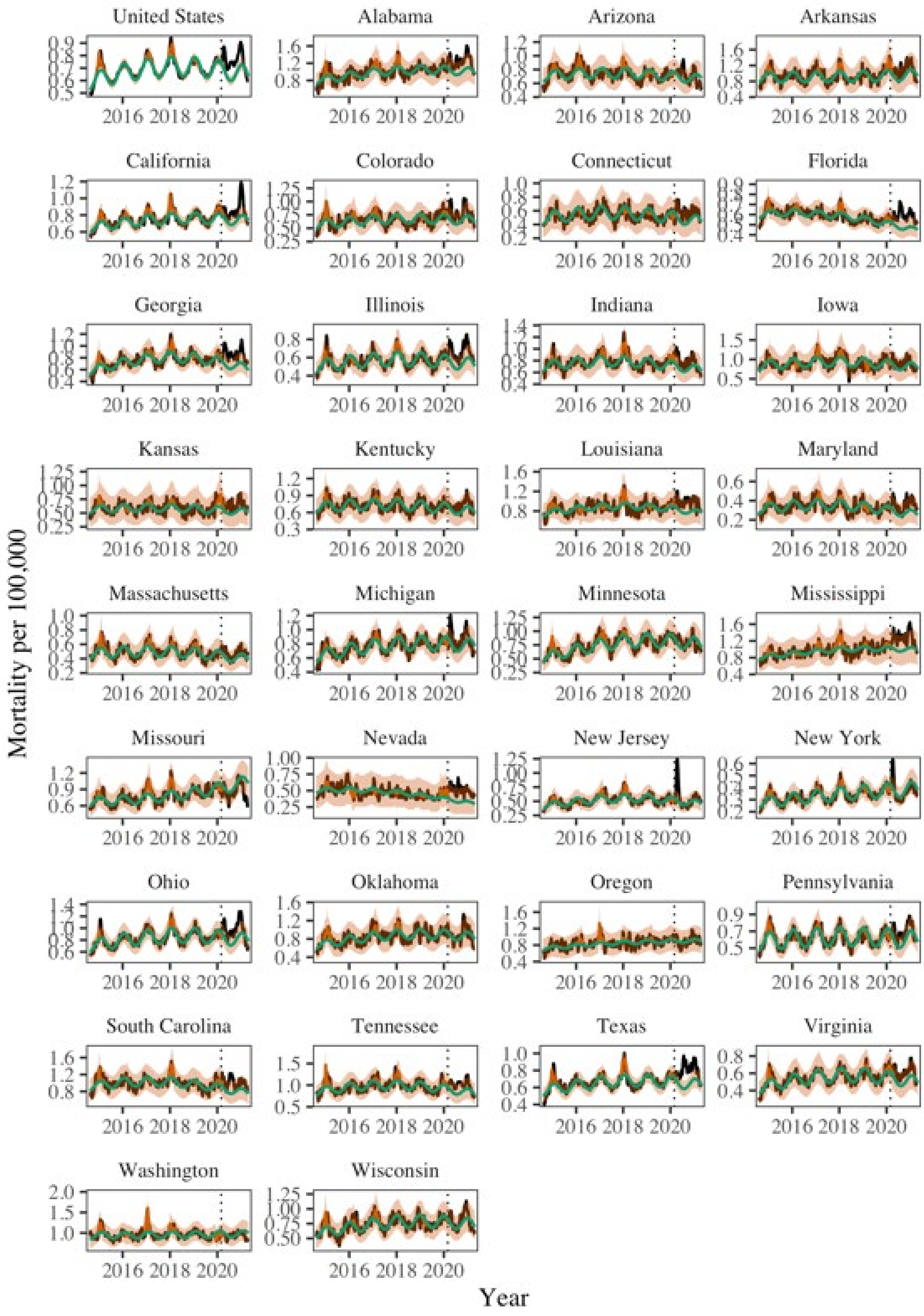

medRxiv preprint doi: https://doi.org/10.1101/2022.02.10.22270721; this version posted February 15, 2022. The copyright holder for this preprint (which was not certified by peer review) is the author/funder, who has granted medRxiv a license to display the preprint in perpetuity.
This article is a US Government work. It is not subject to copyright under 17 USC 105 and is also made available for use under a CC0 license.

Figure S3: Trends in weekly cancer mortality, nationally and by state. Legend as in Figure S1

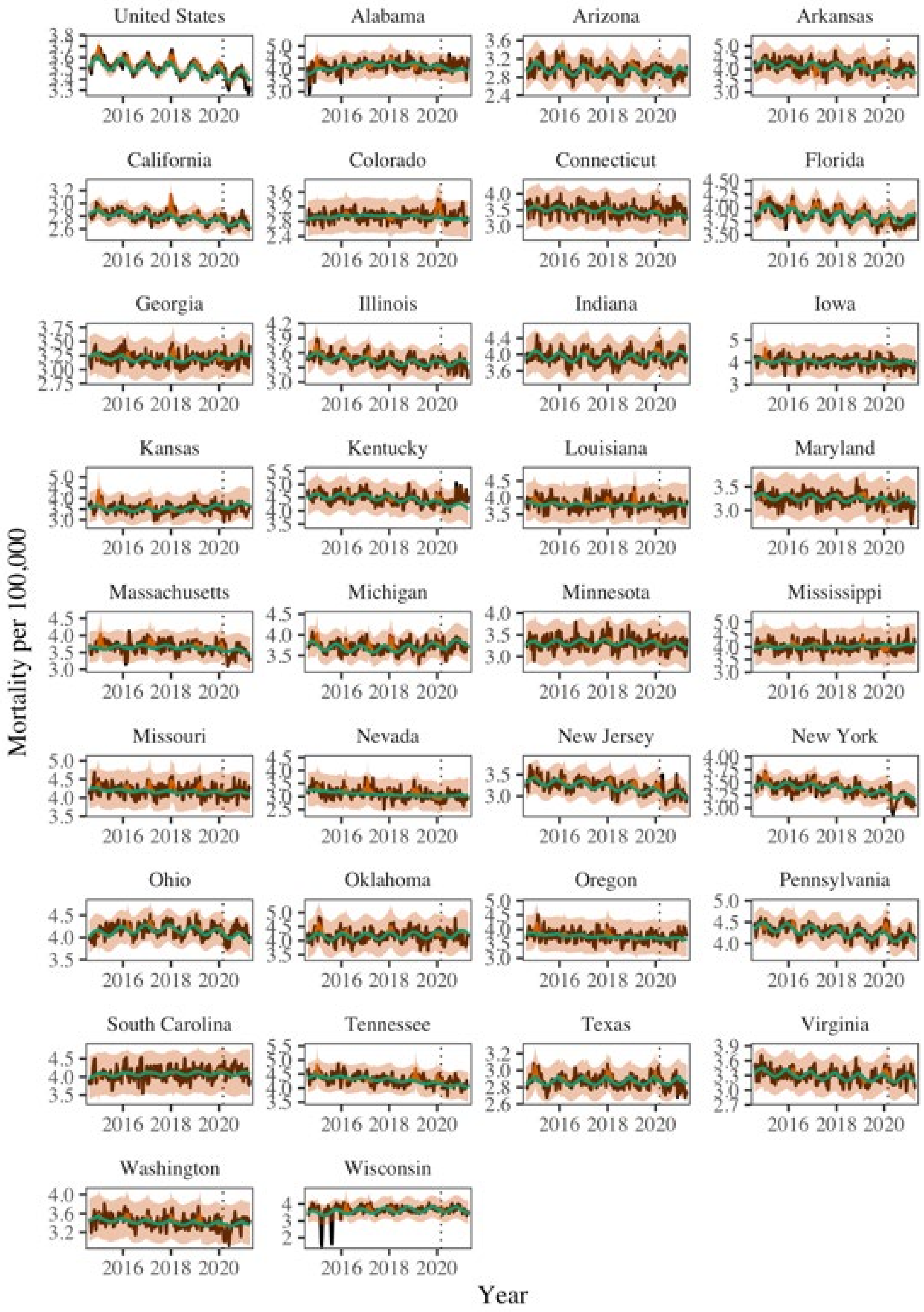

medRxiv preprint doi: https://doi.org/10.1101/2022.02.10.22270721; this version posted February 15, 2022. The copyright holder for this preprint (which was not certified by peer review) is the author/funder, who has granted medRxiv a license to display the preprint in perpetuity.
This article is a US Government work. It is not subject to copyright under 17 USC 105 and is also made available for use under a CC0 license.

Figure S4: Trends in weekly cerebrovascular disease mortality, nationally and by state. Legend as in Figure S1

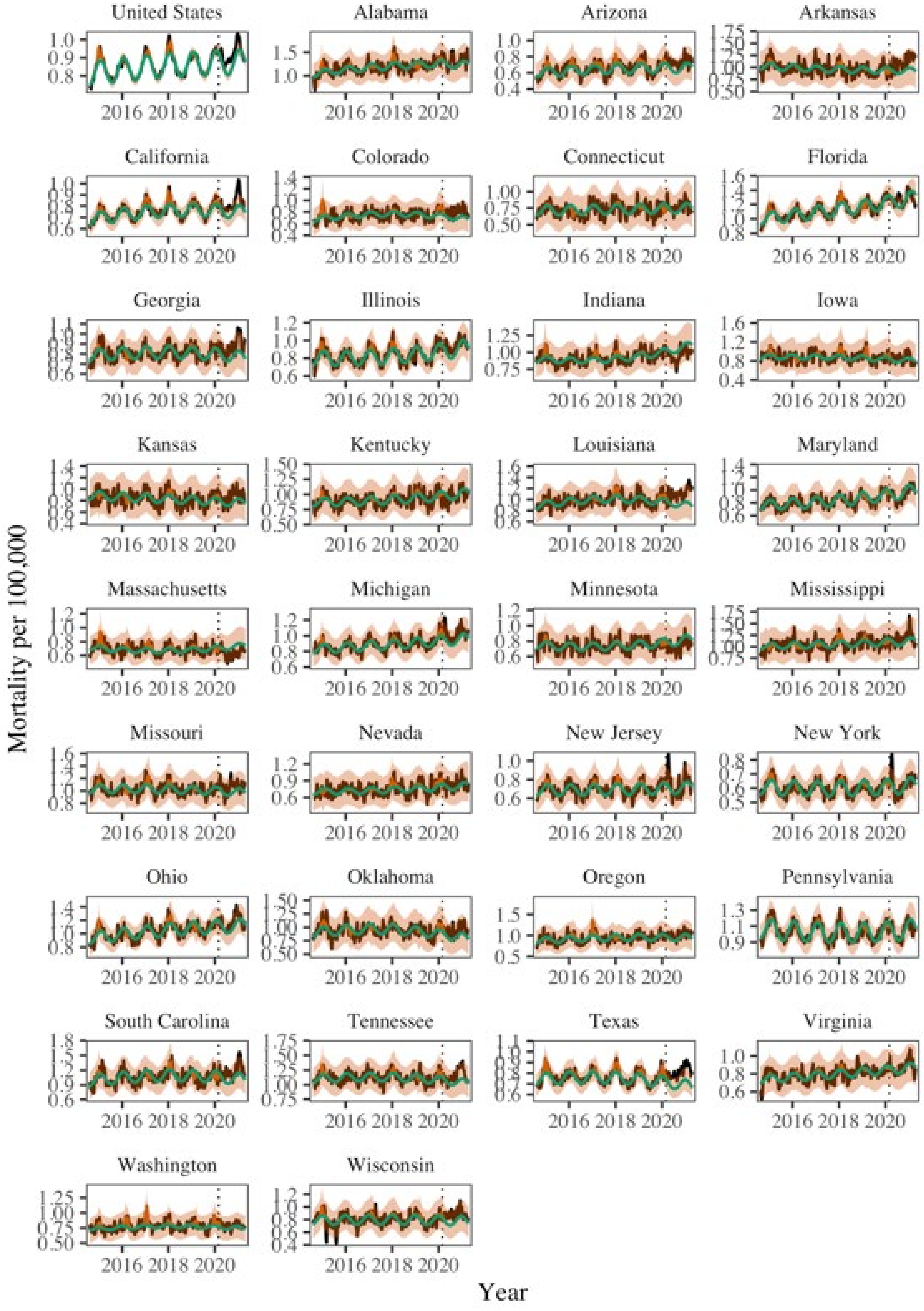

medRxiv preprint doi: https://doi.org/10.1101/2022.02.10.22270721; this version posted February 15, 2022. The copyright holder for this preprint (which was not certified by peer review) is the author/funder, who has granted medRxiv a license to display the preprint in perpetuity.
This article is a US Government work. It is not subject to copyright under 17 USC 105 and is also made available for use under a CC0 license.

Figure S5: Trends in weekly diabetes mortality, nationally and by state. Legend as in Figure S1

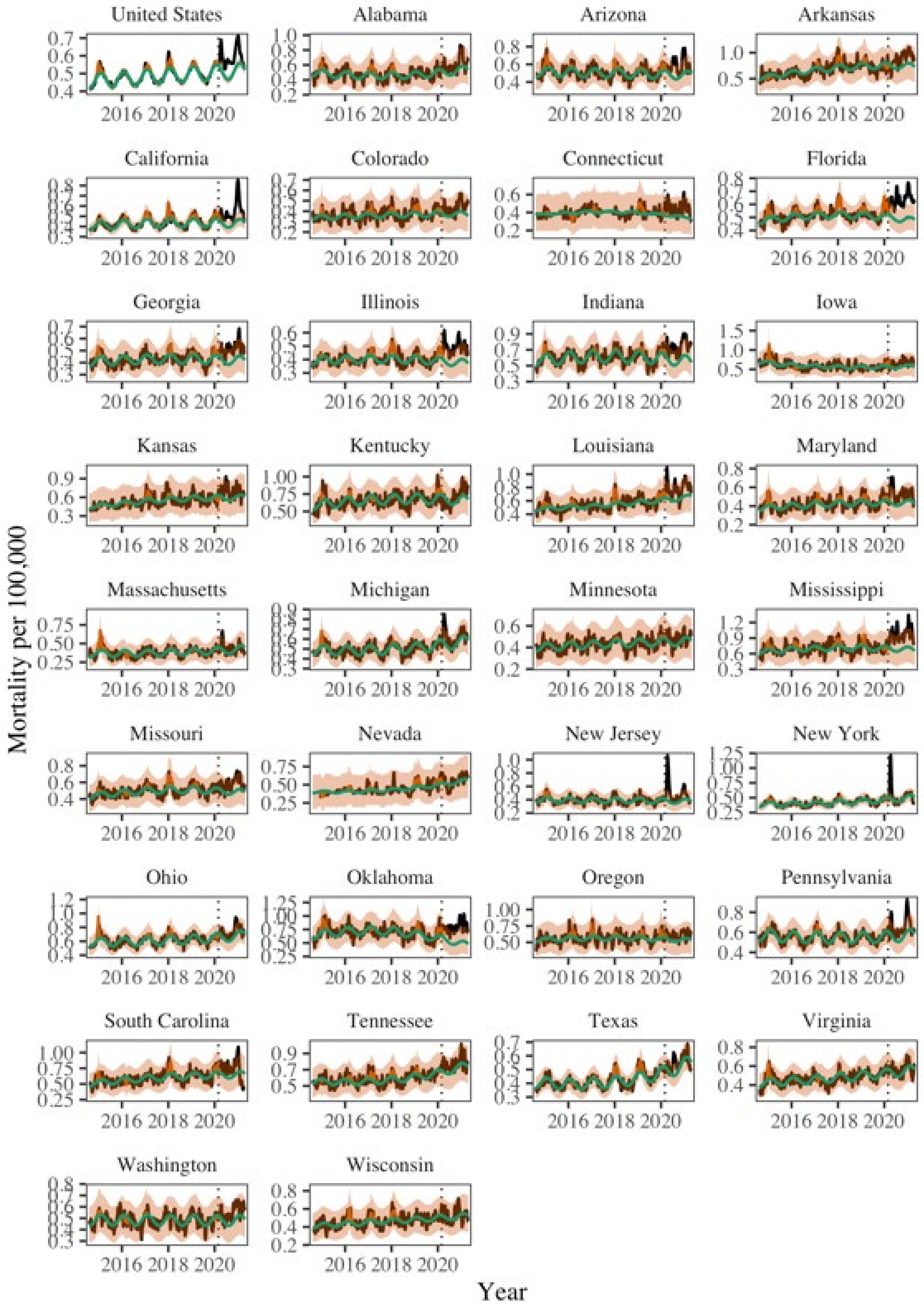

medRxiv preprint doi: https://doi.org/10.1101/2022.02.10.22270721; this version posted February 15, 2022. The copyright holder for this preprint (which was not certified by peer review) is the author/funder, who has granted medRxiv a license to display the preprint in perpetuity.
This article is a US Government work. It is not subject to copyright under 17 USC 105 and is also made available for use under a CC0 license.

Figure S6: Trends in weekly heart disease mortality, nationally and by state. Legend as in Figure S1

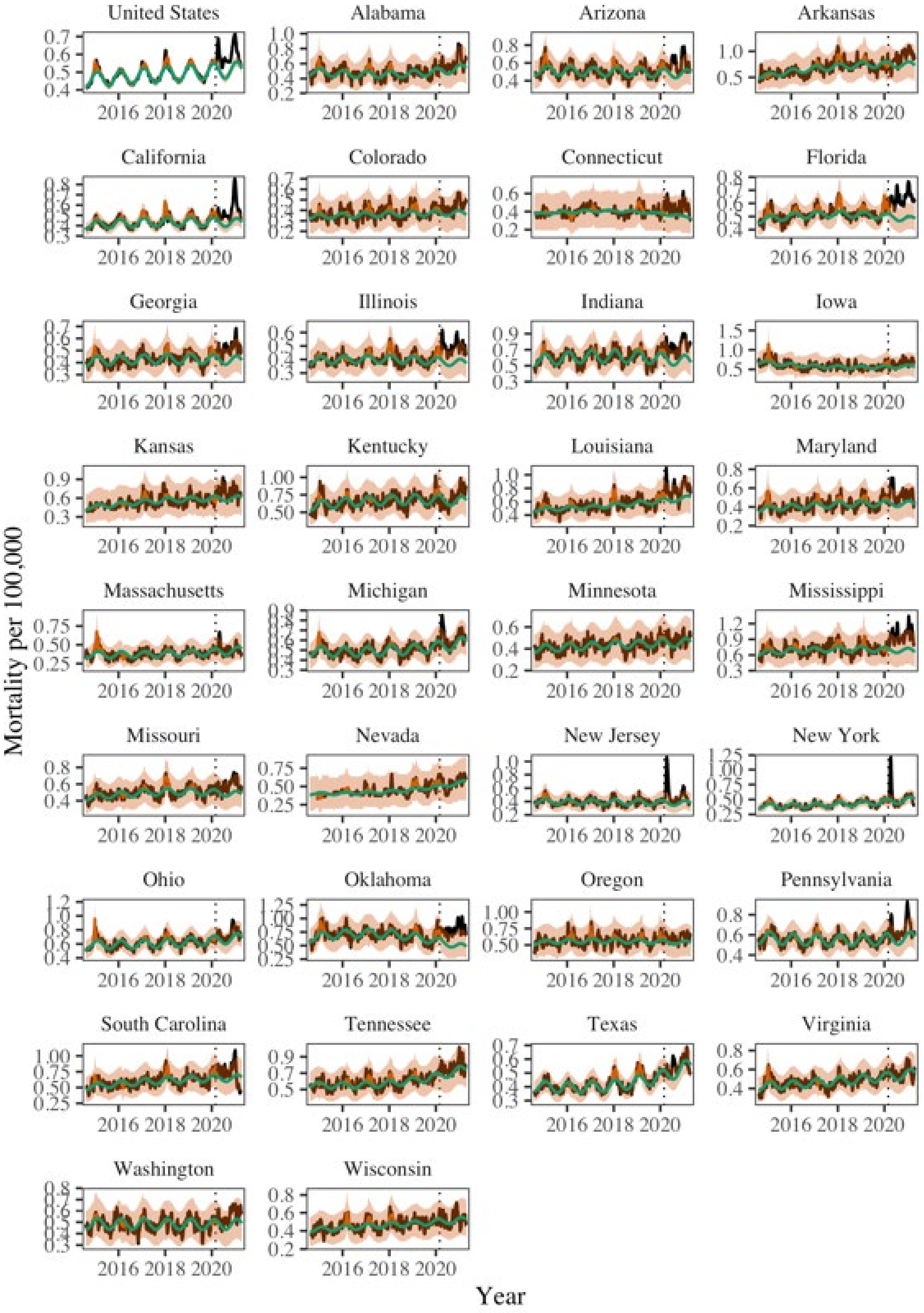

medRxiv preprint doi: https://doi.org/10.1101/2022.02.10.22270721; this version posted February 15, 2022. The copyright holder for this preprint (which was not certified by peer review) is the author/funder, who has granted medRxiv a license to display the preprint in perpetuity.
This article is a US Government work. It is not subject to copyright under 17 USC 105 and is also made available for use under a CC0 license.

Figure S7: Trends in weekly mortality from external causes (opioids, suicides, accidents, etc.), nationally and by state.

Black lines show observed data. Green line shows the seasonal baseline and the red shaded region shows the upper and lower 95% confidence intervals. The dotted vertical line marks March 1, 2020.

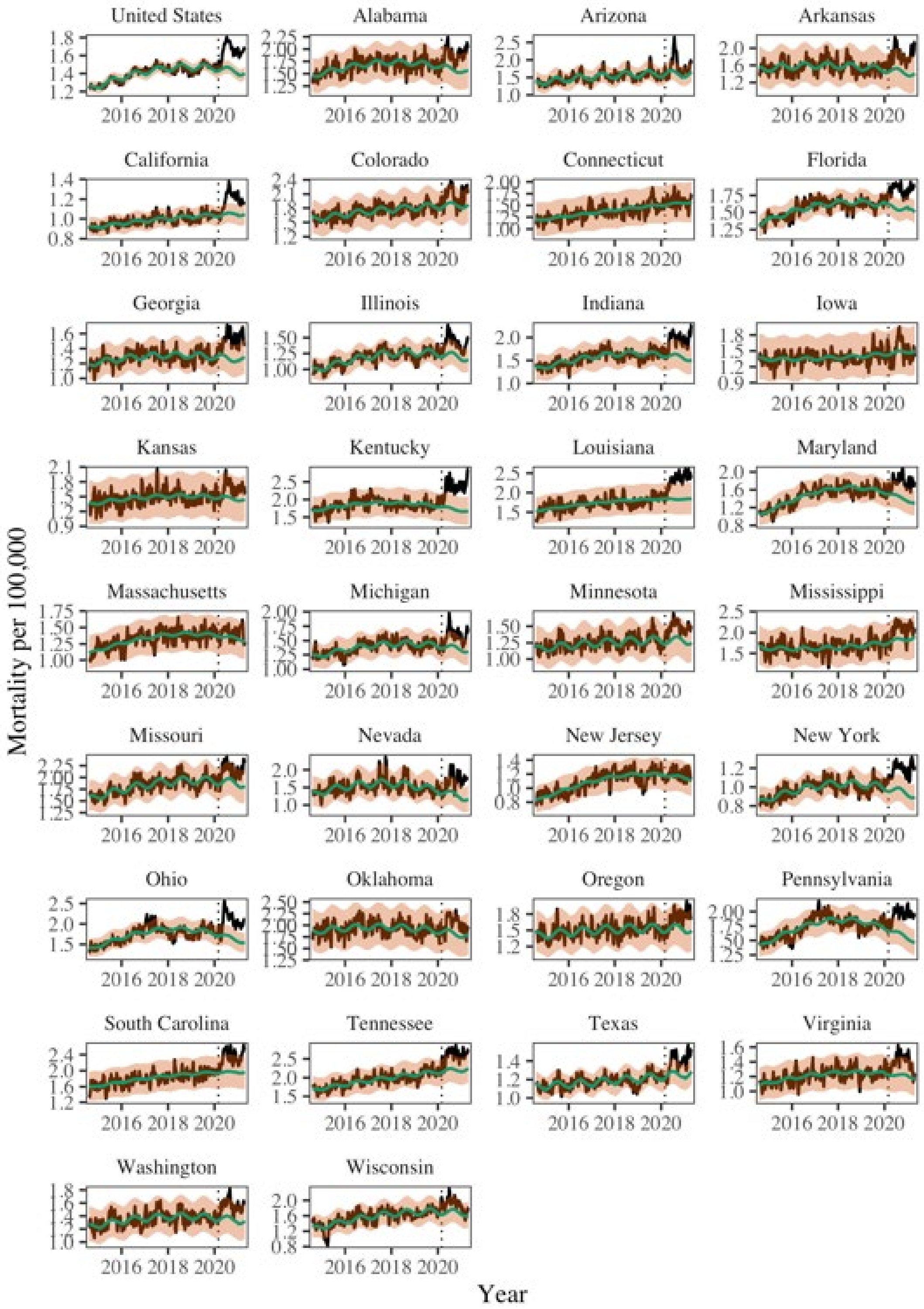

medRxiv preprint doi: https://doi.org/10.1101/2022.02.10.22270721; this version posted February 15, 2022. The copyright holder for this preprint (which was not certified by peer review) is the author/funder, who has granted medRxiv a license to display the preprint in perpetuity. This article is a US Government work. It is not subject to copyright under 17 USC 105 and is also made available for use under a CC0 license.

Figure S8: Trends in weekly respiratory mortality, nationally and by state. Legend as in Figure S1 (only 16 states had sufficient weekly data).

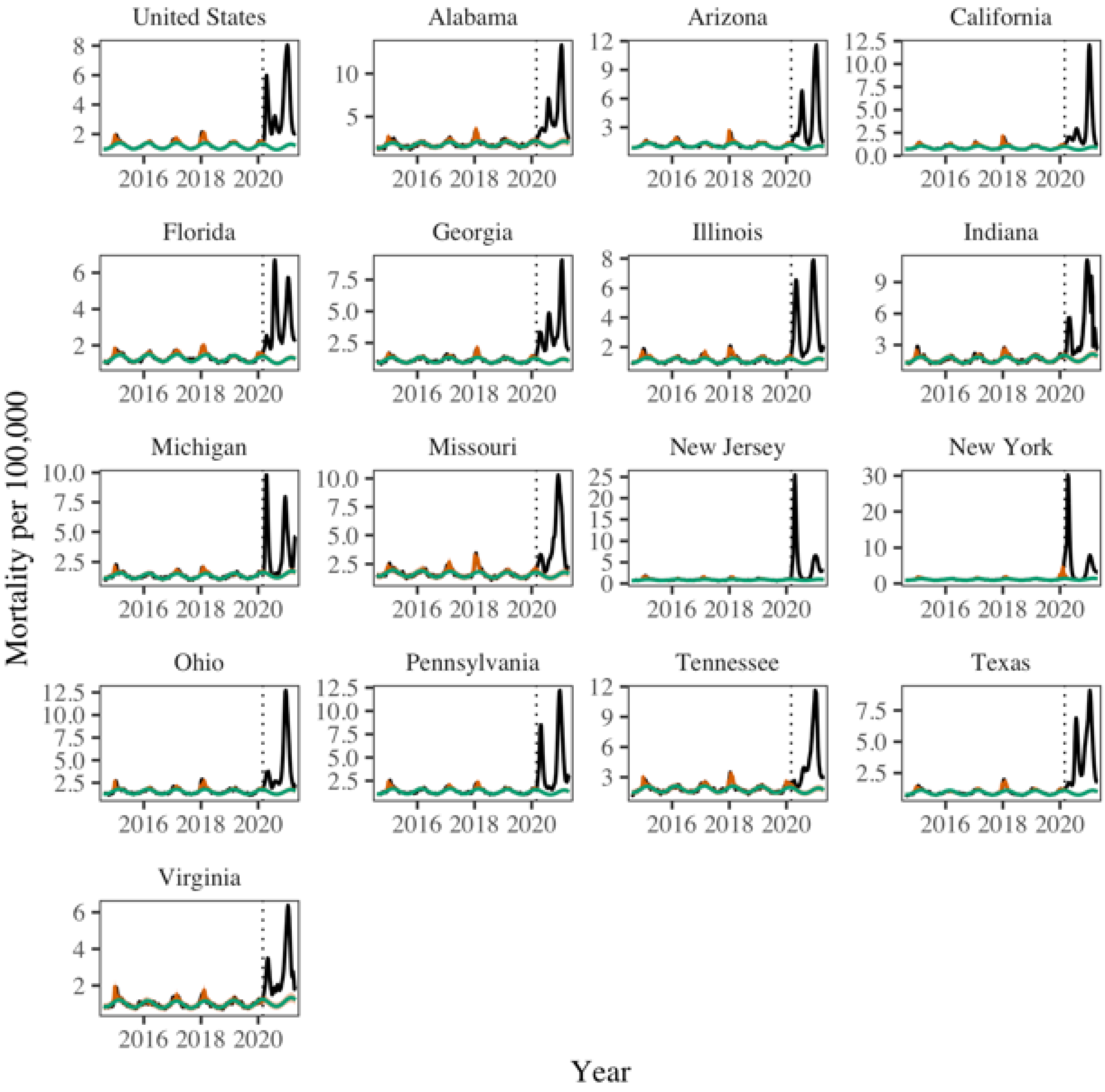

medRxiv preprint doi: https://doi.org/10.1101/2022.02.10.22270721; this version posted February 15, 2022. The copyright holder for this preprint (which was not certified by peer review) is the author/funder, who has granted medRxiv a license to display the preprint in perpetuity.
This article is a US Government work. It is not subject to copyright under 17 USC 105 and is also made available for use under a CC0 license.

Figure S9: Sensitivity Analyses of Infection Fatality Rate (deaths/infections). In sensitivity analyses, we considered different indicators of COVID19 mortality in numerator (excess respiratory deaths, excess all-cause deaths or official COVID-19 death counts), and infection estimates based on seroprevalence in denominator (seroprevalence at the end of the study period, or maximum over the study period), and different age groups (all ages or over 65 yrs, both in numerator and denominator). COVID-19 seroprevalence estimates are from the last week of April 2021. Each point corresponds to a state. States with particularly high infection fatality ratios are annotated. Error bars represent 95% confidence intervals. The black line and dotted region represent a linear regression fit and the associated 95% confidence interval. For instance, using official COVID19 tallies yields an IFR of 0.72% (0.62 – 0.81%).

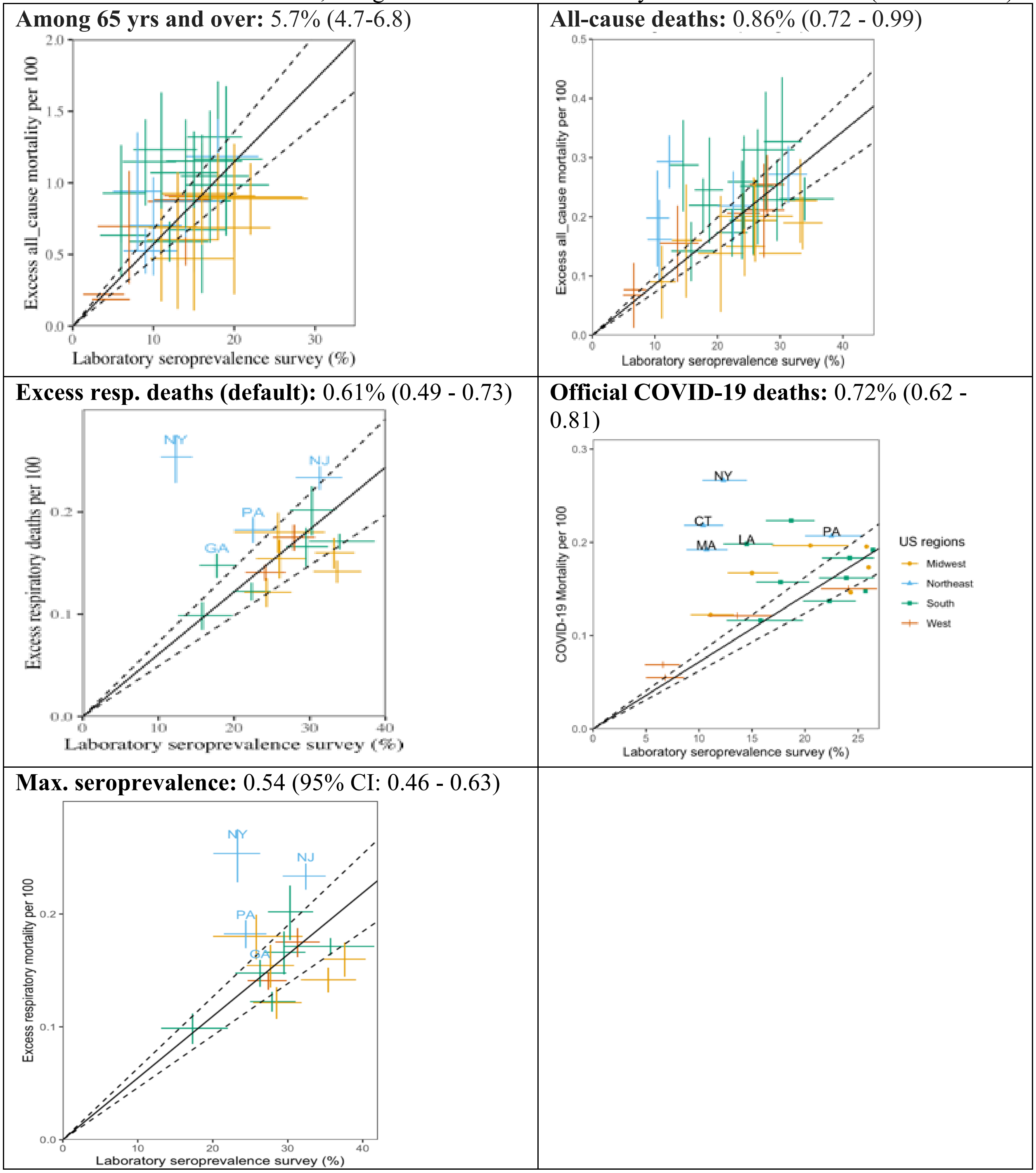

medRxiv preprint doi: https://doi.org/10.1101/2022.02.10.22270721; this version posted February 15, 2022. The copyright holder for this preprint (which was not certified by peer review) is the author/funder, who has granted medRxiv a license to display the preprint in perpetuity.
This article is a US Government work. It is not subject to copyright under 17 USC 105 and is also made available for use under a CC0 license.

Figure S10: Synchrony between respiratory and non-respiratory mortality patterns on a national scale

The black lines show the time-series for each non-respiratory mortality cause. The red line is respiratory mortality. The dotted black lines mark the dates of the peaks during the first, second, and third wave in respiratory mortality (April 18, 2020; August 1, 2020; January 9, 2021).

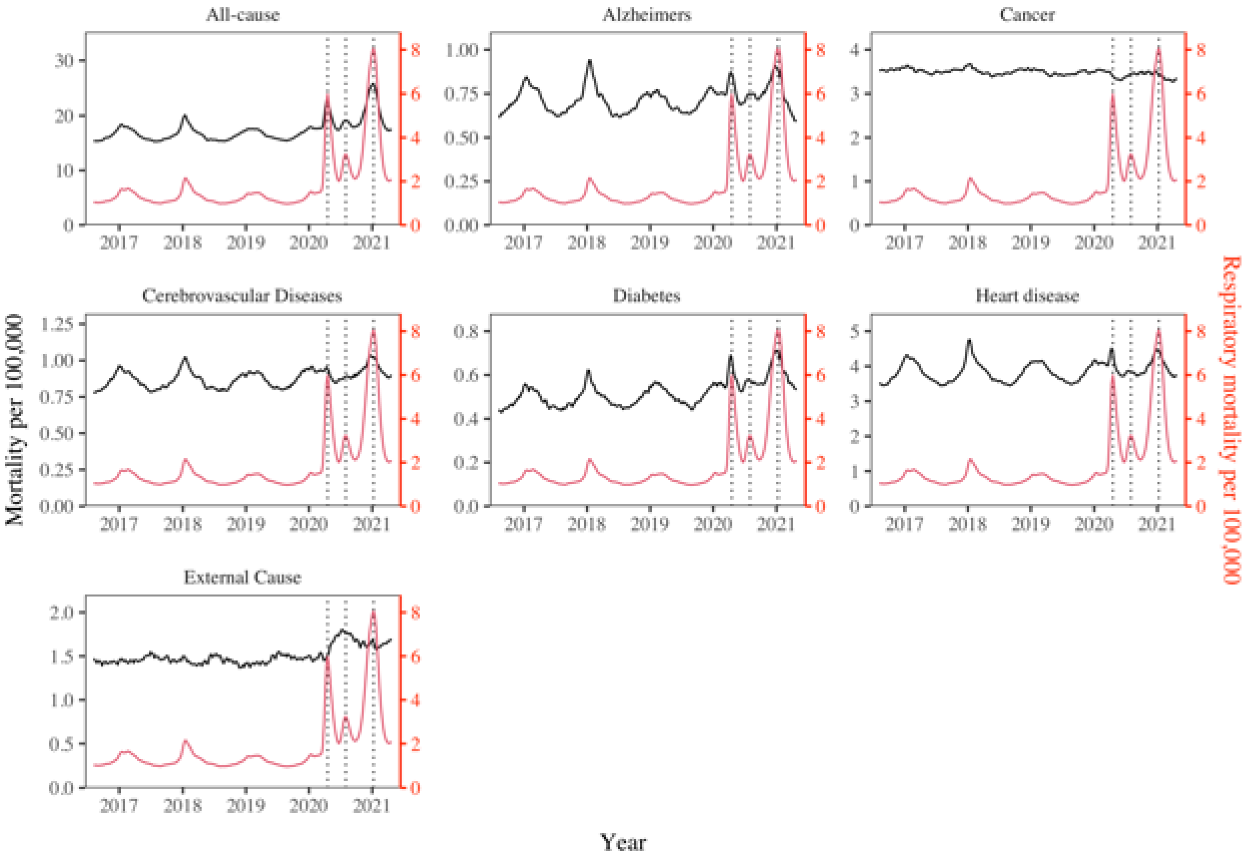

medRxiv preprint doi: https://doi.org/10.1101/2022.02.10.22270721; this version posted February 15, 2022. The copyright holder for this preprint
(which was not certified by peer review) is the author/funder, who has granted medRxiv a license to display the preprint in perpetuity.
This article is a US Government work. It is not subject to copyright under 17 USC 105 and is also made available
for use under a CC0 license.

Figure S11: Correlation between cumulative excess death rates due to external causes (opioids, suicide, accidents, etc.) during March 2020-April 2021 and baseline death rates of external causes, across 33 states. A moderate correlation in these data indicates that states that typically have high rates of mortality from external causes fared worse during the pandemic.

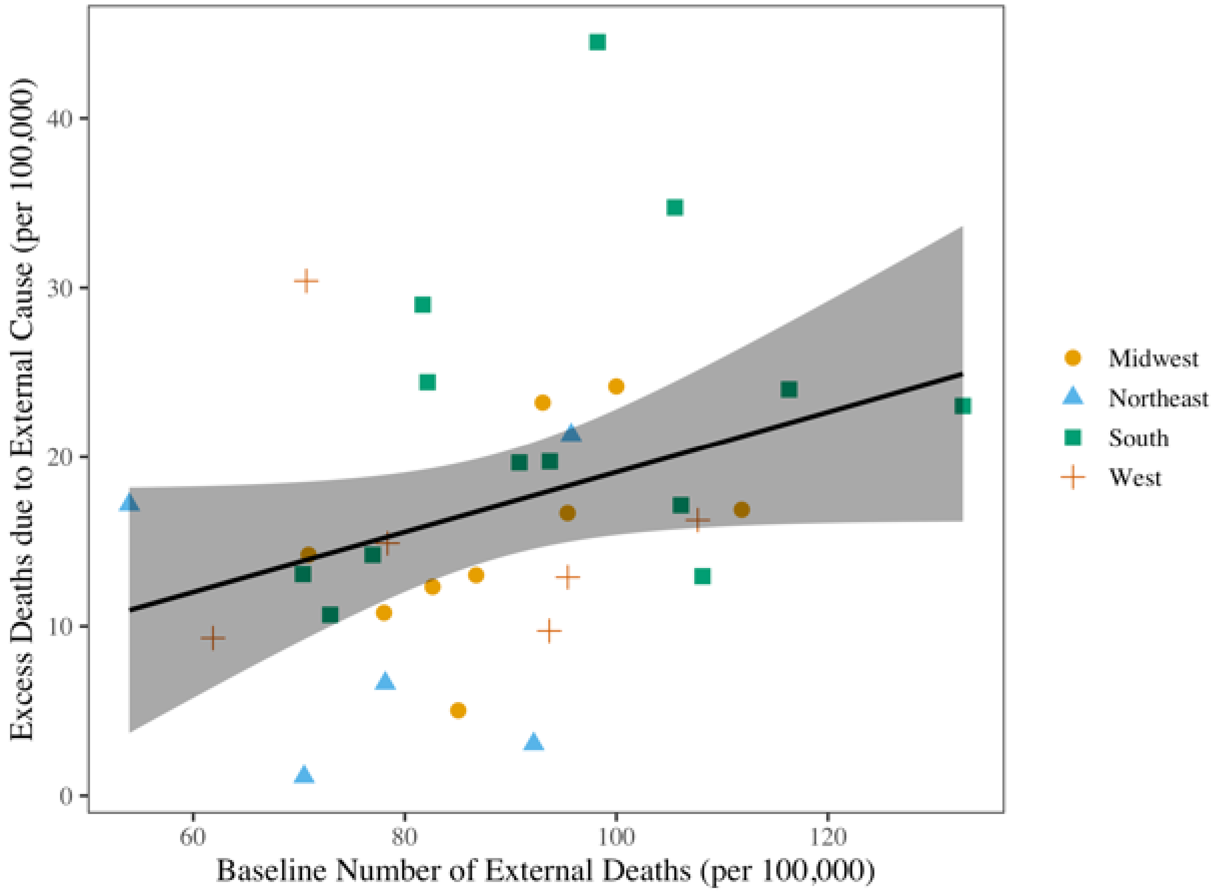

medRxiv preprint doi: https://doi.org/10.1101/2022.02.10.22270721; this version posted February 15, 2022. The copyright holder for this preprint (which was not certified by peer review) is the author/funder, who has granted medRxiv a license to display the preprint in perpetuity.
This article is a US Government work. It is not subject to copyright under 17 USC 105 and is also made available for use under a CC0 license.

Figure S12. Changes in weekly synchrony between respiratory and non-respiratory mortality during the pandemic. Graph compares correlations during 60 weeks of any baseline pre-pandemic period and in the 60 weeks of the pandemic**.** Black points represent estimated pre-pandemic correlations (60 weeks selected before March 2020 by block of 2 weeks). Black error bars represent 95% bootstrap confidence intervals accounting for multiple comparisons using Bonferroni correction. Triangles represent estimated pandemic correlations. Red color indicates significant deviation from pre-pandemic correlation. Correlation is highest for all-cause and is more pronounced during the pandemic period (red triangle), which suggests a direct impact of the virus on these conditions.

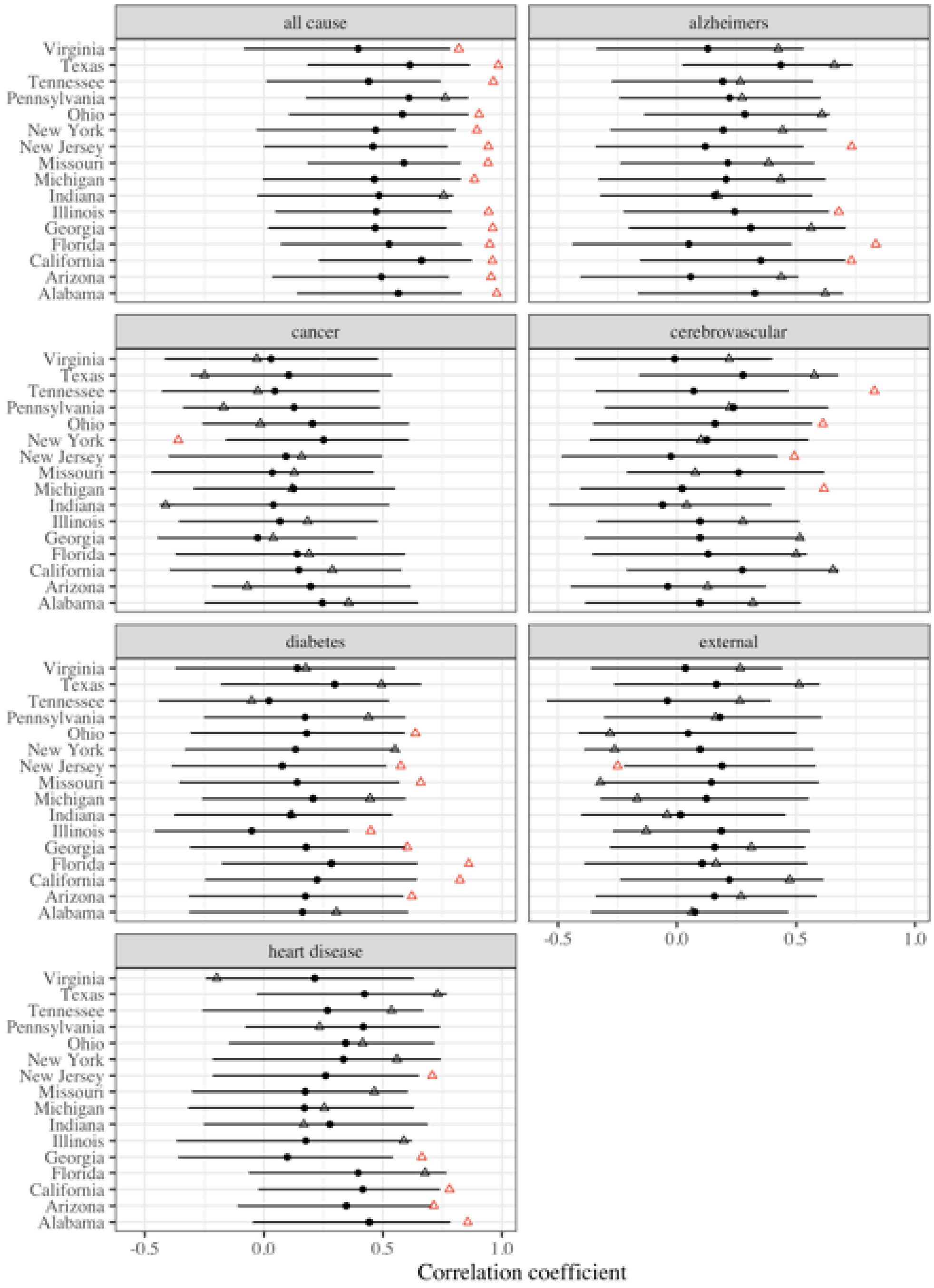

medRxiv preprint doi: https://doi.org/10.1101/2022.02.10.22270721; this version posted February 15, 2022. The copyright holder for this preprint (which was not certified by peer review) is the author/funder, who has granted medRxiv a license to display the preprint in perpetuity.
This article is a US Government work. It is not subject to copyright under 17 USC 105 and is also made available for use under a CC0 license.

Figure S13. Rank correlation between total COVID-19 mortality and total excess mortality for other causes, across 33 states

Black lines represent the best fit regression lines. Shaded areas represent the 95% confidence intervals. The states have been categorized into the Midwest, Northeast, South, and West. Respiratory deaths are moderately to highly correlated with all-cause (rho = 0.73, 95% CI: 0.47 - 0.90)).

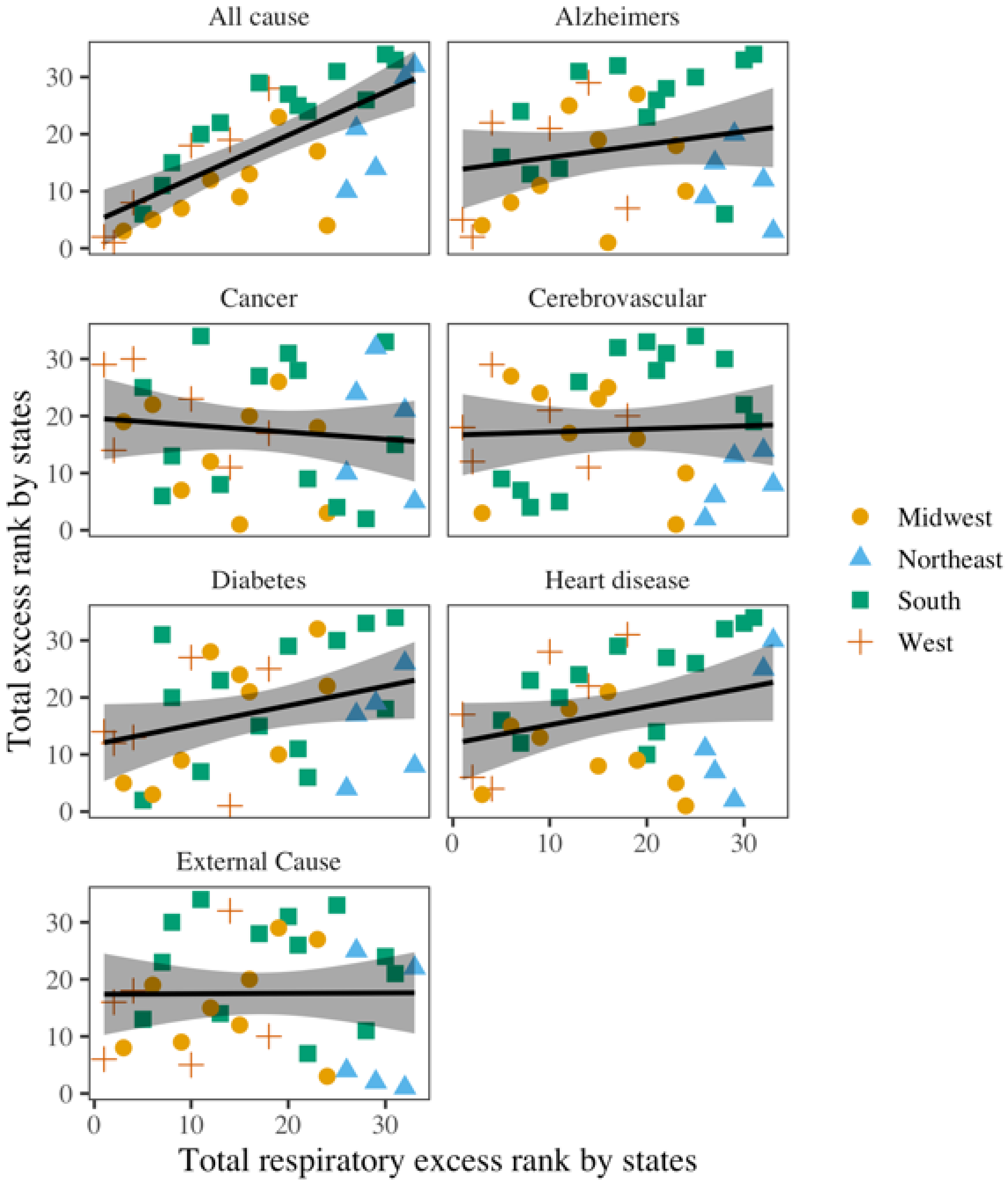

medRxiv preprint doi: https://doi.org/10.1101/2022.02.10.22270721; this version posted February 15, 2022. The copyright holder for this preprint
(which was not certified by peer review) is the author/funder, who has granted medRxiv a license to display the preprint in perpetuity.
This article is a US Government work. It is not subject to copyright under 17 USC 105 and is also made available
for use under a CC0 license.

Figure S14: Monthly number of deaths from external causes by age group, US, January 2019- April 2021.

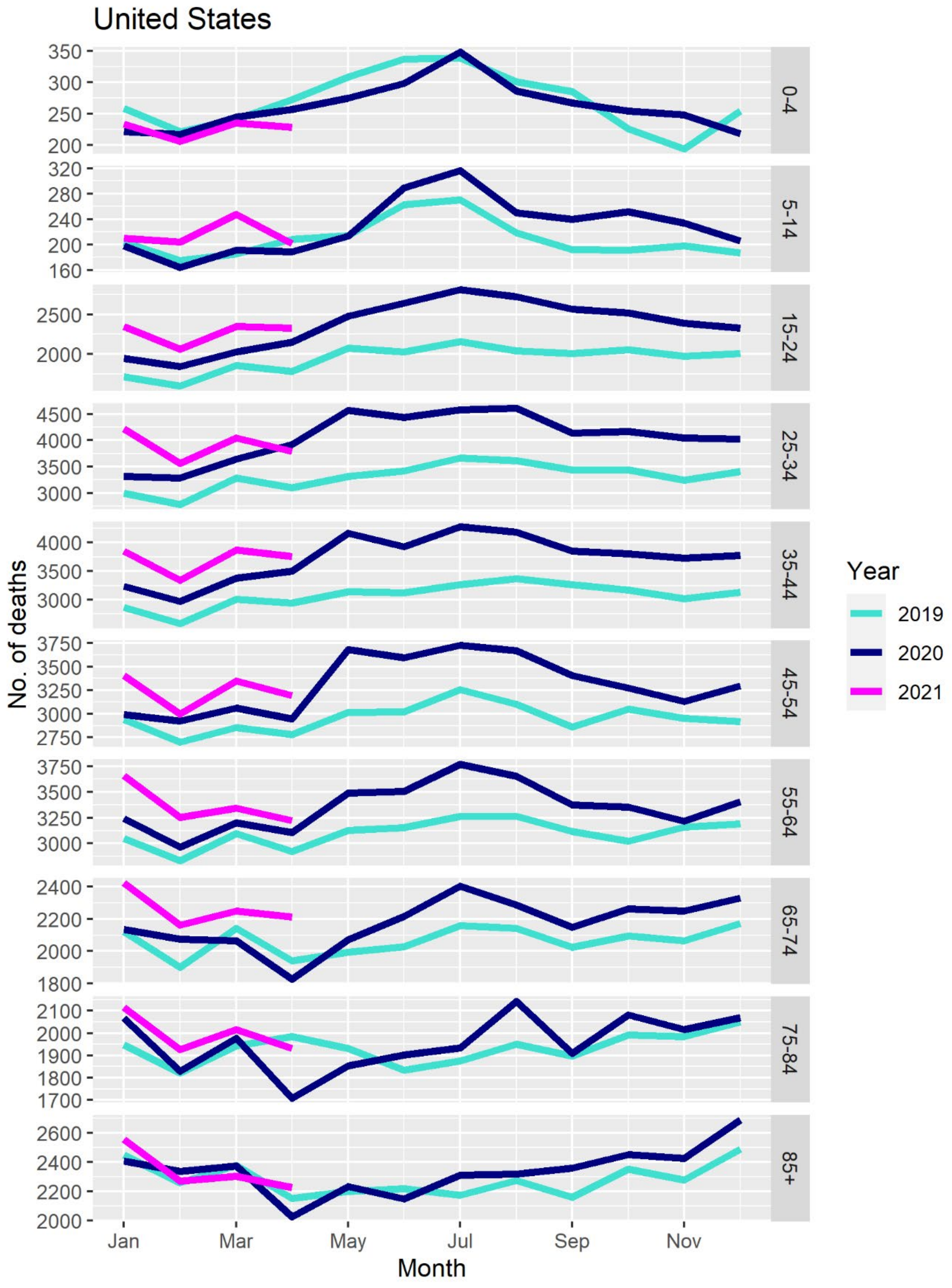

medRxiv preprint doi: https://doi.org/10.1101/2022.02.10.22270721; this version posted February 15, 2022. The copyright holder for this preprint (which was not certified by peer review) is the author/funder, who has granted medRxiv a license to display the preprint in perpetuity. This article is a US Government work. It is not subject to copyright under 17 USC 105 and is also made available for use under a CC0 license.

Supplementary Table 1: Evaluating the direct and indirect impact of the COVID19 pandemic nationally. Regression of national cause-specific weekly excess deaths against interventions and COVID-19 intensity (60 pandemic weeks).

**Cause of Death**	**Univariate**		**Multivariate**	
	COVID-19	Interventions (GRI)	COVID-19	Interventions (GRI)
All Cause	1.158 ± 0.054***	-0.013 ± 0.029	1.157 ± 0.054***	-0.012 ± 0.010
Alzheimer's	0.020 ± 0.005***	-0.001 ± 0.001	0.020 ± 0.005***	
Diabetes	0.018 ± 0.003***	0.000 ± 0.001	0.018 ± 0.003***	
Cancer	-0.001 ± 0.005	-0.004 ± 0.001**		-0.004 ± 0.001**
External Cause	-0.004 ± 0.007	0.007 ± 0.001***	-0.006 ± 0.005	0.007 ± 0.001***
Cerebrovascular	0.008 ± 0.003**	-0.000 ± 0.001	0.008 ± 0.003**	
Heart Disease	0.052 ± 0.016***	-0.003 ± 0.003	0.053 ± 0.016***	-0.004 ± 0.003

*Government response index (GRI) records the stringency of government.
Significance levels: 0-0.001 (***), 0.001-0.01 (**), 0.01-0.05 (*)

medRxiv preprint doi: https://doi.org/10.1101/2022.02.10.22270721; this version posted February 15, 2022. The copyright holder for this preprint (which was not certified by peer review) is the author/funder, who has granted medRxiv a license to display the preprint in perpetuity.
This article is a US Government work. It is not subject to copyright under 17 USC 105 and is also made available for use under a CC0 license.

Supplementary Table 2: Evaluating the direct and indirect impact of the COVID19 pandemic across states. Regression of state- and cause-specific cumulative excess death rates for the period March 2020-April 2021 against average interventions and COVID-19 intensity in the same period and state (33 states).

**Cause of Death**	**Univariate**			**Multivariate**		
	COVID-19	Interventions (GRI)	Pandemic baseline	COVID-19	Interventions (GRI)	Pandemic baseline
All Cause	1.103 ± 0.258***	-0.012 ± 0.035	0.112 ± 0.099	1.147 ± 0.260***	-0.028 ± 0.028	
Alzheimer's	0.025 t± 0.042	-0.003 ± 0.004	-0.010 ± 0.165	0.031 ± 0.043	-0.003 ± 0.004	
Diabetes	0.023 ± 0.035	-0.003 ± 0.004	0.099 ± 0.285	0.029 ± 0.036	-0.003 ± 0.004	
Cancer	-0.006 ± 0.087	0.002 ± 0.009	0.025 ± 0.149	-0.006 ± 0.087	0.002 ± 0.009	0.025 ± 0.149
External Cause	-0.007 ± 0.062	-0.003 ± 0.007	0.196 ± 0.175			0.196 ± 0.175
Cerebrovascular	0.000 ± 0.044	-0.002 ± 0.005	-0.017 ± 0.210		-0.002 ± 0.005	
Heart Disease	0.092 ± 0.100	-0.004 ± 0.011	0.037 ± 0.100	0.092 ± 0.100		

**Government response index (GRI) records the stringency of government interventions. (state cumulative)*

Significance levels: 0-0.001 (***), 0.001-0.01 (**), 0.01-0.05 (*)

medRxiv preprint doi: https://doi.org/10.1101/2022.02.10.22270721; this version posted February 15, 2022. The copyright holder for this preprint (which was not certified by peer review) is the author/funder, who has granted medRxiv a license to display the preprint in perpetuity. This article is a US Government work. It is not subject to copyright under 17 USC 105 and is also made available for use under a CC0 license.

Supplementary Table 3: Evaluating the direct and indirect impact of the COVID19 pandemic by age group. Regression of national age-specific weekly all-cause excess deaths against interventions and COVID-19 intensity (60 pandemic weeks).

**Age Group**	**Univariate**		**Multivariate**	
	COVID-19	Interventions (GRI)	COVID-19	Interventions (GRI)
Under 25 years	0.004 ± 0.002*	5.955 ± 0.880***		5.235 ± 1.534**
25-44 years	0.038 ± 0.005***	28.80 ± 2.610***	0.023 ± 0.003***	22.49 ± 2.142***
45-64 years	0.199 ± 0.010***	77.74 ± 14.38***	0.180 ± 0.009***	29.18 ± 5.611***
65-74 years	0.264 ± 0.008***	73.11 ± 20.23***	0.262 ± 0.009***	2.486 ± 5.625
75-84 years	0.320 ± 0.015***	63.04 ± 26.68	0.338 ± 0.015***	-27.97 ± 9.612**
85 years and older	0.381 ± 0.025***	75.37 ± 33.86*	0.403 ± 0.027***	-33.07 ± 17.27

Significance levels: 0-0.001 (***), 0.001-0.01 (**), 0.01-0.05 (*)
